# Graph limits of random graphs from a subset of connected k‐trees

**DOI:** 10.1002/rsa.20802

**Published:** 2018-09-11

**Authors:** Michael Drmota, Emma Yu Jin, Benedikt Stufler

**Affiliations:** ^1^ Institut für Diskrete Mathematik und Geometrie Technische Universität Wien Vienna Austria; ^2^ Unité de Mathématiques Pures et Appliquées École Normale Suprieure de Lyon Lyon Cedex 07 France

**Keywords:** continuum random tree, modified Galton‐Watson tree, partial k‐trees

## Abstract

For any set Ω of non‐negative integers such that {0,1}⊊Ω, we consider a random Ω‐k‐tree G_n,k_ that is uniformly selected from all connected k‐trees of (n + k) vertices such that the number of (k + 1)‐cliques that contain any fixed k‐clique belongs to Ω. We prove that G_n,k_, scaled by $\left(kH_{k}\sigma_{\Omega })/(2\sqrt{n}\right)$ where H_k_ is the kth harmonic number and σ_Ω_ > 0, converges to the continuum random tree $T_{e}$. Furthermore, we prove local convergence of the random Ω‐k‐tree $G_{n,k}^{\circ }$ to an infinite but locally finite random Ω‐k‐tree G_∞,k_.

## 1 INTRODUCTION AND MAIN RESULTS

**Figure 1:**
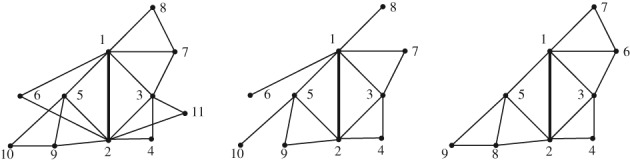
A 2‐tree (left), a partial 2‐tree (middle) and an Ω‐2‐tree (right) where Ω = {0,1,2}

A *k‐tree* is a generalization of a tree and can be defined recursively: it is either a complete graph on *k* vertices (= a *k*‐clique) or a graph obtained from a smaller *k*‐tree by adjoining a new vertex together with *k* edges connecting it to a *k*‐clique of the smaller *k*‐tree (and thus forming a (*k* + 1)‐clique). In particular, a 1‐tree is a usual tree. (Note that the parameter *k* is always fixed.) Subgraphs of *k*‐trees are called *partial k‐trees*; see Figure 1.

A partial *k*‐tree is an interesting graph from an algorithmic point of view since many NP‐hard problems on graphs have polynomial, in fact usually linear, dynamic programming algorithms when restricted to partial *k*‐trees for fixed values of *k* 8, 38, 63; such NP‐hard problems include maximum independent set size, minimal dominating set size, chromatic number, Hamiltonian circuit, network reliability and minimum vertex removal forbidden subgraph^7^, ^13^. Several graphs which are important in practice^48^, have been shown to be partial *k*‐trees, among them are

1. Trees/Forests (partial 1‐trees)
2. Series parallel networks (partial 2‐trees)
3. Outplanar graphs (partial 2‐trees)
4. Halin graphs (partial 3‐trees); see 37.

However, other interesting graph classes like planar graphs or bipartite graphs are not partial *k*‐trees. On the other hand, partial *k*‐trees are very interesting from a combinatorial point of view, although the enumeration of partial *k*‐trees for general *k* is still missing. The *k*‐trees are the maximal graphs with treewidth ≤*k*, in the sense that adding another edge would increase the treewidth. The number of *k*‐trees has been counted in various ways; see 11, 18, 29, 30, 32, 34, 40, 41, 56. As usual a graph on *n* vertices is called *labelled* if the integers from {1,2,…,*n*} are assigned to its vertices (one‐to‐one).

In this paper, we introduce a subset of connected labelled *k*‐trees, called *Ω‐k‐trees*, as a first attempt to approach the profile of connected labelled partial *k*‐trees by using the enumeration of labelled *k*‐trees. In what follows, without specifying otherwise, we assume that Ω‐*k*‐trees are all labelled and a random Ω‐*k*‐tree is uniformly selected from the class of labelled Ω‐*k*‐trees with (*n* + *k*) vertices.

Definition 1 (Ω‐*k*‐tree). For any set Ω of non‐negative integers which contains 0,1 and at least one integer greater than 1, an Ω‐*k*‐tree is a connected *k*‐tree such that the number of (*k* + 1)‐cliques that contain any fixed *k*‐clique belongs to the set Ω.

A rooted Ω‐*k*‐tree is an Ω‐*k*‐tree rooted at a *k*‐clique. If $\Omega =N_{0}=\{0,1,2,\cdots \}$, an $N_{0}$‐*k*‐tree is a *k*‐tree. See Figure 1 for an example of an Ω‐2‐tree. We remark that it is necessary to allow 0 ∈ Ω since by construction a *k*‐clique is the smallest *k*‐tree. We also need the condition 1 ∈ Ω. Otherwise any *k*‐tree, other than a single *k*‐clique, is infinite. We disregard the case Ω = {0,1} in order to consider just non‐trivial classes.

Darrasse and Soria 18 proved that the expected distance between pairs of vertices in a random *k*‐tree with *n* vertices converges to a Rayleigh distribution after rescaling it by $1/\sqrt{n}$. The Rayleigh distribution also arises as the distance between typical vertices in Aldous' *continuum random tree* (CRT)  3, 4, 5. This motivates the question whether the classical convergence of 1‐trees to the CRT may be extended to the case *k* ≥ 2 and to models of random partial *k*‐trees.

Since Aldous's pioneering work on the Galton‐Watson trees, the CRT has been established as the limiting object of a large variety of combinatorial structures 12, 14, 16, 17, 39, 44, 54, 57, 58, 61. A key idea in the study of these combinatorial objects is to relate them to trees endowed with additional structures by using an appropriate bijection. In the present case of Ω‐*k*‐trees, we encode them as so‐called (*k*,Ω)‐coding trees via a bijection due to Darrasse and Soria 18, which was originally used to enumerate *k*‐trees and to recursively count the distance between any two vertices in a random *k*‐tree. Furthermore, in order to build a connection between the distance of two vertices in a random Ω‐*k*‐tree and the distance of two vertices in a critical Galton‐Watson tree, we introduce the concept of a *size‐biased* (*ξ*_•_,*ξ*_∘_)*‐multitype Galton‐Watson tree*. This is adapted from the *size‐biased Galton‐Watson tree* which was defined by Kesten 47, used by Lyons and coworkers in 52, by Addario‐Berry, Devroye and Janson in 1, and was further generalized to the *size‐biased* R‐*enriched tree* by Panagiotou and coworkers 58.

When we analyze Ω‐*k*‐trees, it turns out that it is convenient to consider the number of *hedra* instead of the number of vertices as the size of an Ω‐*k*‐tree (we adopt the notions from 34). A *hedron* is a (*k* + 1)‐clique in an Ω‐*k*‐tree, and by definition an Ω‐*k*‐tree with *n* hedra has (*n* + *k*) vertices. A *front* of a *k*‐tree is a *k*‐clique.

Our first main result establishes the convergence of a random *k*‐tree to the CRT with respect to the Gromov‐Hausdorff‐Prokhorov distance.

Theorem 1 *Let* $G_{n,k}$ *be the class of labelled Ω‐k‐trees with n hedra and denote by* G_*n*,*k*_ *a random Ω‐k*‐*tree that is uniformly selected from the class* $G_{n,k}$. *Let* $\mu_{G_{n,k}}$ *be the uniform measure on the set of vertices of* $G_{n,k}$. *Then*

$$\begin{array}{lcr} \left(G_{n,k},\frac{kH_{k}\sigma_{\Omega }}{2\sqrt{n}}{\text{dist}}_{G_{n,k}},\mu_{G_{n,k}})\overset{d}{\underset{\;}{\to }}(T_{e},d_{e},\mu_{e}\right) & & \end{array}$$

*holds with respect to the Gromov‐Hausdorff‐Prokhorov metric. Here* ${\text{dist}}_{G_{n,k}}$ *is the graph distance of* G_*n*,*k*_, *H*_*k*_ = 1 + 1/2 + … + 1/*k denotes the kth harmonic number and σ*_Ω_ *is a positive constant. If* $\Omega =N_{0}$, *then the constant* $\sigma_{N_{0}}$ *equals 1*.

This theorem shows in particular that the diameter and the distance of two independently selected random vertices in a random Ω‐*k*‐tree G_*n*,*k*_ are of order $\sqrt{n}$, and that up to scaling factors we obtain the same limiting distribution as that for random 1‐trees. In fact, by general properties of the Gromov‐Hausdorff‐Prokhorov metric[55, Prop. 10], this statement may be extended to the case of any constant number of independent and uniform random vertices. Our proofs also show that the diameter of the random *k*‐tree G_*n*,*k*_ may be stochastically bounded by the diameter of a critical Galton‐Watson tree conditioned to be large, where the offspring distribution has finite exponential moments. Hence the corresponding tail‐bounds for the height of large Galton‐Watson trees  1 allow us to deduce arbitrarily large uniform integrability for the rescaled diameter and rescaled distance of two random vertices in G_*n*,*k*_. Together with the distributional limits, this yields precise asymptotic expressions for all moments.

Instead of the class $G_{n,k}$, we could equivalently consider the class of Ω‐*k*‐trees with *n* hedra that are rooted at a fixed labelled front. In section 2.1 we will argue that the two models are equivalent and hence our results apply to both.

We recall that (partial) 1‐trees are just trees and partial 2‐trees are series‐parallel graphs. In both cases it is known 5, 57 that the CRT appears as the scaling limit (if we scale the metric by $c/\sqrt{n}$ for some positive constant *c*). We conjecture that the CRT also arises as the scaling limit of partial *k*‐trees for larger *k*. At the moment, this property seems to be out of reach, since there is no precise asymptotic analysis of partial *k*‐trees if *k* ≥ 3.

Conjecture 1 Let ${PT}_{n,k}$ be the class of all connected labelled partial *k*‐trees and let PT_*n*,*k*_ be a uniform random graph from ${PT}_{n,k}$. Then, for every *k* ≥ 1, PT_*n*,*k*_ converges toward the CRT in the Gromov‐Hausdorff‐Prokhorov sense, after rescaling the metric by a factor $c_{k}/\sqrt{n}$ for some constant *c*_*k*_ > 0.

Theorem 1 describes the asymptotic global metric properties of random *k*‐trees, but gives little information about asymptotic local properties. Hence we provide a second limit theorem that establishes local weak convergence of the random Ω‐*k*‐tree G_*n*,*k*_ toward an infinite but locally finite Ω‐*k*‐tree G_*∞*,*k*_. This type of convergence describes the asymptotic behaviour of neighborhoods around a uniform random front.

Theorem 2 *Let* $G_{n,k}^{\circ }$ *be the random front‐rooted* Ω‐*k‐tree that is obtained by marking a uniform random front of the random* Ω‐*k‐tree* G_*n*,*k*_. *Then, as n tends to infinity, the random graph* $G_{n,k}^{\circ }$ *converges in the local‐weak sense toward a front‐rooted infinite* Ω‐*k‐tree* G_*∞*,*k*_, *that is,*

$$G_{n,k}^{\circ }\overset{d}{\underset{\;}{\to }}G_{\infty ,k}.$$

Our proof of Theorem 2 builds on the classical local convergence of simply generated trees toward a modified Galton‐Watson tree. See for example Theorem 7.1 in Janson's survey 42, which unifies some results by Kennedy 46, Aldous and Pitman 6, Grimmett 36, Kolchin 49, Kesten 47, Aldous 4, Jonsson and Stefánsson 45 and Janson, Jonsson and Stefánsson 43.

A result similar to Theorem 2 is known for neighborhoods of random vertices in partial 2‐trees since series‐parallel graphs belong to the family of subcritical graph classes 33, 60, 62. This motivates the following conjecture.

Conjecture 2 For every *k* ≥ 1, the random labelled partial *k*‐tree PT_*n*,*k*_ converges in the local‐weak sense. That is, the neighborhoods of a random vertex in PT_*n*,*k*_ converge weakly toward the neighborhoods of an infinite rooted partial *k*‐tree *PT*_*∞*,*k*_ as *n* tends to infinity.

The plan of the paper is as follows. In Section 2 we recall the combinatorial background for Ω‐*k*‐trees, introduce a multitype Galton‐Watson process in order to uniformly generate a random (*k*,Ω)‐coding tree, describe Darrasse and Soria's algorithm to compute the distances between two vertices in an Ω‐*k*‐tree, present Aldous's result on the convergence of critical Galton‐Watson trees to the CRT $T_{e}$, and recall the notion of local convergence. In Section 3 we prove our first main result—Theorem 1, and in Section 4 our second main result—Theorem 2.

## 2 COMBINATORICS, MULTITYPE GALTON‐WATSON TREES AND GRAPH LIMITS

For any integer *i* ≥ 0, we set [*i*]: = {1,2,…,*i*}. Let $\Omega \subset N_{0}$ denote a set of non‐negative integers which contains 0,1 and at least one integer greater than 1. We will review the generating function approach from 18 to count the number of Ω‐*k*‐trees. The key ingredient is a bijection between rooted Ω‐*k*‐trees and *(k,Ω)‐coding trees*.

Definition 2 ((*k*,Ω)‐coding tree). For any set Ω of non‐negative integers which contains 0,1 and at least one integer greater than 1, a *(k,Ω)‐coding tree* of size *n* is a tree *T* consisting of *kn* + 1 white nodes and *n* black nodes which satisfies the following conditions:

1. *T* is rooted at a white node. Every white node has an unordered list of black nodes as children and every black node has an ordered list of precisely *k* white nodes as children.
2. The number of black children of the white root belongs to the set Ω and the number of black children of any other white one lies in the shifted set
  $$\Omega_{\text{out}}=\{i\;|\;i+1\in \Omega ,i\geq 0\}.$$
3. The white root of *T* is labelled by a strictly increasing sequence (*a*_1_,*a*_2_,…,*a*_*k*_) where *A* = {*a*_1_,*a*_2_,…,*a*_*k*_} is a *k*‐subset of [*n* + *k*]. The black nodes are labelled by the integers from the set [*n* + *k*]∖*A*.
4. We label non‐root white nodes of *T* recursively: if a black node is labelled with *r* and it is a child of a white node labelled with the sequence (*r*_1_,*r*_2_,…,*r*_*k*_), then starting from the left, the *i*th child of the black node *r* is labelled with the sequence (*r*_1_,…,*r*_*i* − 1_,*r*,*r*_*i* + 1_,…,*r*_*k*_), which is a sequence obtained from (*r*_1_,*r*_2_,…,*r*_*k*_) by replacing *r*_*i*_ by *r*.

In this way the labels on the white root and on the black nodes determine the labels on the non‐root white nodes. If the white root of a (*k*,Ω)‐coding tree *C* has precisely one black child, we call *C* a *reduced (k,Ω)‐coding tree*.

The following classes and random graphs will play a key role in our arguments:

1. $G_{n,k}$: the class of labelled Ω‐*k*‐trees with *n* hedra.
2. G_*n*,*k*_: a random Ω‐*k*‐tree that is uniformly selected from the class $G_{n,k}$.
3. $G_{n,k}^{\circ }$: the class of labelled Ω‐*k*‐trees with *n* hedra that are rooted at a front.
4. $G_{n,k}^{\circ }$: a random Ω‐*k*‐tree G_*n*,*k*_ that is rooted at a uniformly chosen front. This is equivalent to uniformly selecting an element from $G_{n,k}^{\circ }$.
5. $G_{n,k}^{□}$: the class of labelled Ω‐*k*‐trees with *n* hedra that are rooted at a fixed front [*k*].
6. $G_{n,k}^{•}$: the class of labelled Ω‐*k*‐trees with *n* hedra that are rooted at a fixed front [*k*] and this root front is contained in only one hedron.
7. $C_{n,k}$: the class of (*k*,Ω)‐coding trees with *n* black vertices, such that the white root is labelled with (1,2,…,*k*).
8. *C*_*n*,*k*_: a random (*k*,Ω)‐coding tree that is uniformly selected from $C_{n,k}$.
9. $B_{n,k}$: the class of reduced (*k*,Ω)‐coding trees with *n* black vertices, such that the white root is labelled with (1,2,…,*k*).
10. *B*_*n*,*k*_: a random reduced (*k*,Ω)‐coding tree that is uniformly selected from $B_{n,k}$.
11. $G_{n,k}^{•}$: a random Ω‐*k*‐tree that uniquely corresponds to *B*_*n*,*k*_ under the bijection *ϕ* from section 2.2. This is equivalent to choosing a random Ω‐*k*‐tree uniformly from the class $G_{n,k}^{•}$.
12. $G_{n,k}^{□}$: a random Ω‐*k*‐tree that uniquely corresponds to *C*_*n*,*k*_ under the bijection *ϕ*. This is equivalent to choosing a random Ω‐*k*‐tree uniformly from the class $G_{n,k}^{□}$.

### 2.1 Comparison of different rooting procedures

Any Ω‐*k*‐tree with *n* hedra has precisely *kn* + 1 fronts. Hence it makes no difference whether we uniformly select an element from the class $G_{n,k}$ or from the class $G_{n,k}^{\circ }$ of labelled Ω‐*k*‐trees with *n* hedra that are rooted at a front. Instead of studying the random graph G_*n*,*k*_ (as in Theorem 1) or the random graph $G_{n,k}^{\circ }$ (as in Theorem 2), it suffices to consider uniformly selected elements from the class $G_{n,k}^{\circ }$.

We can even make another simplification: For any *k*‐subset $A=\{a_{1},a_{2},\cdots ,a_{k}\}\subset [n+k]$ we may consider the subset $M_{A}\subset G_{n,k}^{\circ }$ of elements where the vertices of the root‐front are labelled with *a*_1_,*a*_2_,…,*a*_*k*_. Given any other *k*‐subset $A^{\prime }\subset [n+k]$ we can choose a bijection *f*:[*n* + *k*]→[*n* + *k*] with *f*(*A*) = *A* ^*′*^. Let $\psi_{f}:G_{n,k}^{\circ }\to G_{n,k}^{\circ }$ denote the corresponding relabelling function, that permutes the labels on the vertices according to *f*. As $\psi_{f}(M_{A})\subset M_{A^{\prime }}$ and $\psi_{f}(M_{A^{\prime }})\subset M_{A}$, it follows that *ψ*_*f*_ induces a bijection from *M*_*A*_ to $M_{A^{\prime }}$.

Thus, $G_{n,k}^{\circ }$ is the disjoint union of n+kk‐many relabelled versions of the class $G_{n,k}^{□}:=M_{\left[k\right]}$, where the root‐front is required to be labelled from 1 to *k*. Hence, instead of studying G_*n*,*k*_ or $G_{n,k}^{\circ }$, it suffices to study uniform elements from the class $G_{n,k}^{□}$.

### 2.2 Correspondence between k‐trees and coding trees

There is a bijection

$$\phi :G_{n,k}^{□}\to C_{n,k}$$

between the class $G_{n,k}^{□}$ of front‐rooted Ω‐*k*‐trees with *n* hedra (where the root‐front is labelled from 1 to *k*) and the class $C_{n,k}$ of all (*k*,Ω)‐coding trees with *n* black vertices where the white root is labelled with the sequence (1,2,…,*k*).

**Figure 2:**
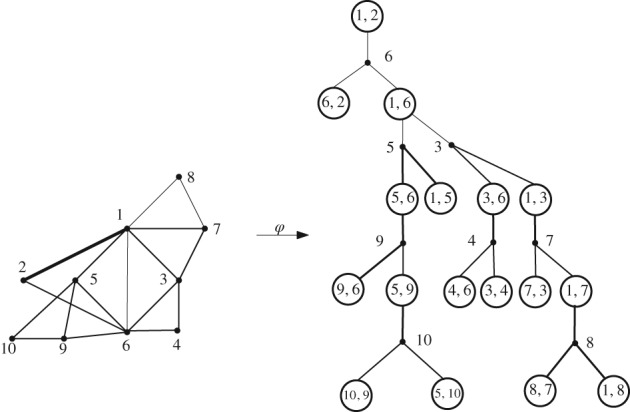
Example of the correspondence between an Ω‐2‐tree (where Ω = {0,1,2,3}) with front labels 1,2 (left) and a (2,Ω)‐coding tree C_n,2_ rooted at a white node labelled by (1,2) (right)

The correspondence *𝜑* is defined such that black nodes in a (*k*,Ω)‐coding tree correspond to hedra in a Ω‐*k*‐tree. Every black node also gets a label which is equal to the label of one of the vertices of the corresponding hedron. A white node in a (*k*,Ω)‐coding tree corresponds to a front of the Ω‐*k*‐trees and is labelled by the strictly increasing sequence (*a*_1_,*a*_2_,…,*a*_*k*_) of labels of the corresponding front. A black node is a child of a white node if the corresponding hedron contains the corresponding front and the label of the black node is just the label of the vertex that is not contained in the front. Thus, if we start with the root‐front of the Ω‐*k*‐tree, we can recursively build up a corresponding (*k*,Ω)‐coding tree; see Figure 2.

With the help of this correspondence *𝜑*, the problem of counting Ω‐*k*‐trees with *n* hedra is reduced to counting the corresponding (*k*,Ω)‐coding trees with *n* black nodes. In what follows we use the notation *∘‐rooted (k,Ω)‐coding trees* if the white root has a fixed label and use the notation ∘ − • (*k,Ω*)‐*coding tree* if the white root of a reduced (*k*,Ω)‐coding tree has a fixed label.

Let $G_{k}^{□}$ be the class of Ω‐*k*‐trees rooted at a fixed front {1,2,…,*k*}, $C_{k}$ be the class of (*k*,Ω)‐coding trees, and $B_{k}$ be the class of ∘ − • (*k*,Ω)‐coding trees. Clearly the correspondence *𝜑* also establishes a bijection $\phi :G_{k}^{□}\to C_{k}$. The next goal is to formulate a recursive structure of $C_{k}$ and $B_{k}$, where we make use of the standard construction rules for labelled combinatorial objects 28. In this language every (*k*,Ω)‐coding tree can be identified as a set of ∘ − • (*k*,Ω)‐coding trees with the outdegree set Ω:

(1) $$\begin{array}{ll} C_{k}={\text{SET}}_{\Omega }(B_{k}). & \; \end{array}$$

In terms of exponential generating functions (where the size is always the number of black nodes), we thus get

(2) $$\begin{array}{ll} C_{k}(z)=\sum_{i\in \Omega }\frac{{\left(B_{k}(z)\right)}^{i}}{i\;!}. & \; \end{array}$$

We continue to decompose ∘ − • (*k*,Ω)‐coding tree. Let $C_{k}^{\circ }$ be the class of ∘‐rooted (*k*,Ω)‐coding trees that are contained in the ∘ − • (*k*,Ω)‐coding trees. Then every tree from $C_{k}^{\circ }$ can be identified as a set of ∘ − • (*k*,Ω)‐coding trees with the outdegree set Ω_out_ = {*i*|*i* + 1 ∈ Ω,*i* ≥ 0} of the white nodes. Moreover, every ∘ − • (*k*,Ω)‐coding tree can be decomposed into a *k*‐tuple of ∘‐rooted (*k*,Ω)‐coding trees. This yields the following specification:

(3) $$B_{k}=\{•\}*{\text{SEQ}}_{k}(C_{k}^{\circ })\;\;\;\text{and}\;\;C_{k}^{\circ }={\text{SET}}_{\Omega_{\text{out}}}(B_{k}).$$

In terms of exponential generating functions, one gets

(4) $$B_{k}(z)=z\cdot C_{k}^{\circ }{\left(z\right)}^{k}\;\;\;\text{and}\;\;C_{k}^{\circ }(z)=\sum_{\begin{array}{c} i+1\in \Omega \\ i\geq 0 \end{array}}\frac{{\left(B_{k}(z)\right)}^{i}}{i\;!}.$$

In particular B_*k*_(*z*) satisfies

(5) $$B_{k}(z)=z{\left(\sum_{\begin{array}{c} i+1\in \Omega \\ i\geq 0 \end{array}}\frac{{\left(B_{k}(z)\right)}^{i}}{i\;!}\right)}^{k}.$$

By applying standard theory (see 28) it follows that B_*k*_(*z*) has finite radius of convergence *ρ*_*k*,Ω_ and has also the property that B_*k*_(*ρ*_*k*,Ω_) is finite. In particular the value of B_*k*_(*ρ*_*k*,Ω_) is given by the relation

(6) $$\begin{array}{ll} \sum_{\begin{array}{c} i+1\in \Omega \\ i\geq 0 \end{array}}\frac{\left(ki-1\right)}{i!}{\left(B_{k}(\rho_{k,\Omega })\right)}^{i}=0. & \; \end{array}$$

In principle we have to distinguish between the *aperiodic case*, where all coefficients of B_*k*_(*z*) are positive and *ρ*_*k*,Ω_ is the only singularity on the radius of convergence |*z*| = *ρ*_*k*,Ω_, and the *periodic case*, where B_*k*_(*z*) has several singularities on its radius |*z*| = *ρ*_*k*,Ω_, see 9. In the periodic case the coefficients [*z* ^*n*^]B_*k*_(*z*) are positive precisely for *n* = 1 mod*d*, where $d=\gcd(\Omega_{\text{out}})$. In both cases we obtain asymptotics for [*z* ^*n*^]B_*k*_(*z*), see Lemma 8 and Theorem 3 in 9. So for the sake of simplicity, we assume that we are working in the *aperiodic case*. That is, [*z* ^*n*^]B_*k*_(*z*) has a unique dominant singularity *z* = *ρ*_*k*,Ω_.

It follows immediately from (2) and (4) that *C*_*k*_(*ρ*_*k*,Ω_) < *∞* and $C_{k}^{\circ }(\rho_{k,\Omega })<\infty$. We set *b*_*k*,Ω_(*n*) = *n*![*x* ^*n*^]B_*k*_(*x*) and *c*_*k*,Ω_(*n*) = *n*![*x* ^*n*^]*C*_*k*_(*x*). One can analyze the asymptotic behaviors of *b*_*k*,Ω_(*n*) and *c*_*k*,Ω_(*n*) from the recursive equation (5), see 20, 28. This yields

(7) $$\begin{array}{ll} b_{k,\Omega }(n)∼d_{1}n^{-3/2}n!\rho_{k,\Omega }^{-n}\;\text{and}\;c_{k,\Omega }(n)∼d_{2}n^{-3/2}n!\rho_{k,\Omega }^{-n} & \; \end{array}$$

for some positive constants *d*_1_,*d*_2_. In order to obtain exact expressions for *b*_*k*,Ω_(*n*) we may apply the Lagrange inversion formula to Eq. 5. For the case $\Omega =N_{0}$ this yields

(8) $$b_{k,N_{0}}(n)=(n-1)![x^{n-1}]\exp(knx)={\left(kn\right)}^{n-1}$$

and

(9) $$c_{k,N_{0}}(n)=(n-1)![x^{n-1}]\exp((kn+1)x)={\left(kn+1\right)}^{n-1}={\left(kn+1\right)}^{n-1}.$$

Hence the number of (*k*,Ω)‐coding trees having an arbitrarily labelled root is given by

n+kk(kn+1)n−2,

which is consistent with the classical enumeration of *k*‐trees  11, 18, 29, 56.

### 2.3 Multitype Galton‐Watson tree

In order to generate (uniformly) a ∘ − • (*k*,Ω)‐coding tree we make use of a multitype Galton‐Watson process. Let *ξ*_∘_ be an integer‐valued random variable with probability distribution

(10) $$\begin{array}{ll} P[\xi_{\circ }=i] & =\frac{1}{C_{k}^{\circ }(\rho_{k,\Omega })}\frac{{\left(B_{k}(\rho_{k,\Omega })\right)}^{i}}{i\;!}\;\text{if}\;i\in \Omega_{\text{out}}\;\;\text{and}\;\;P[\xi_{\circ }=i]=0\;\text{otherwise}.\; \end{array}$$

Furthermore let *ξ*_•_ be another integer‐valued random variable with probability distribution

(11) $$\begin{array}{ll} P[\xi_{•}=k] & =1\;\;\text{and}\;\;P[\xi_{•}=i]=0\;\text{if}\;i\neq k.\; \end{array}$$

The (*ξ*_•_,*ξ*_∘_)‐*multitype Galton‐Watson tree* M is then given by the family tree of a Galton‐Watson branching process with alternating offspring distributions *ξ*_•_ and *ξ*_∘_. We start with a black node •, which gives birth to a set of white nodes ∘ according to offspring distribution *ξ*_•_, and each white node ∘ gives birth to a set of black nodes • according to offspring distribution *ξ*_∘_. We denote by |M| the number of black nodes of M.

Every (*ξ*_•_,*ξ*_∘_)‐multitype Galton‐Watson tree generated by this process is a plane tree, where the children of each node (white or black) are equipped with a *left‐to‐right* order. For simplicity, we assume that the white root of any ∘ − • (*k*,Ω)‐coding tree is always labelled with the sequence (1,2,…,*k*).

In Lemma 3 we consider all different labellings of a (*ξ*_•_,*ξ*_∘_)‐multitype Galton‐Watson tree, where the labels on the white non‐root nodes are determined by the labels on the black nodes and the white root. Two labellings are regarded as *different* if the edge sets are different.

Lemma 3 *The following procedure terminates almost surely:*

1. *Draw a* (*ξ*_•_,*ξ*_∘_)‐*multitype Galton‐Watson tree* M.
2. *Choose a uniform random labelling of* M *such that all black nodes of* M *are labelled with distinct integers* (k+1),(k+2),⋯,(k+|M|).
3. *Add a white root to* M *and label it with the sequence* (1,2,…,*k*).

*This procedure draws a random* ○ − • (*k*,Ω)‐*coding tree* B_*k*_ *according to a critical Boltzmann distribution. That is, for every* $B\in B_{n,k}$ (*of size n* ≥ 1) *we have*

$$\begin{array}{ll} P[B_{k}=B]=\frac{{\left(\rho_{k,\Omega }\right)}^{n}}{n!\;B_{k}(\rho_{k,\Omega })}. & \; \end{array}$$

**Figure 3:**
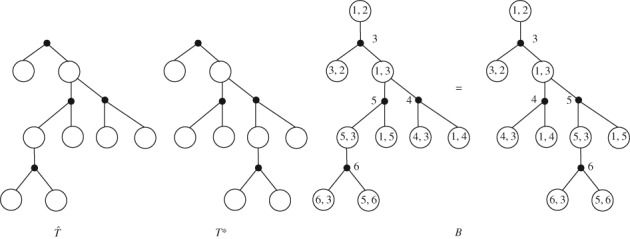
$\hat{T}$ and T ^∗^ are two different (ξ_•_,ξ_∘_)‐multitype Galton‐Watson trees after step (1), which lead to the same ∘ − • (k,Ω)‐coding tree B after steps (2) and (3) in Lemma 3

Note that different (*ξ*_•_,*ξ*_∘_)‐multitype Galton‐Watson trees after step (1) could lead to the same ∘ − • (*k*,Ω)‐coding tree after steps (2) and (3); see Figure 3.

Let *B* be any ∘ − • (*k*,Ω)‐coding tree of size *n* and let *T* be the underlying unlabelled (*k*,Ω)‐coding tree of *B* after we remove the white root and the edge connecting the white root.

We denote by E(T) the set of embeddings of *T* into the plane where only the children of *white* nodes are equipped with a left‐to‐right order. Though every embedding of *T* is a (*ξ*_•_,*ξ*_∘_)‐multitype Galton‐Watson tree, only when applying the steps (2) and (3) on two different embeddings from the subset E(T) can we possibly generate the same (*k*,Ω)‐coding tree *B*; see Figure 3 for an example. The essential reason is the set‐relation in (3), where only the children of every white node in *B* are equipped with the set‐relation (without the left‐to‐right order).

If *T* has *n* black nodes, i.e., |*T*| = *n*, then *T* has *kn* white nodes. Suppose that the outdegree sequence of these *kn* white nodes is (*d*_1_,*d*_2_,…,*d*_*kn*_) where *d*_*i*_ ∈ Ω_out_. Clearly, *d*_1_ + *d*_2_ + ⋯ + *d*_*kn*_ = *n* − 1. By combining (10), (11) and (4), we find that every (*ξ*_•_,*ξ*_∘_)‐multitype Galton‐Watson tree M that is an embedding of *T* in E(T) is drawn with probability

$$\begin{array}{ll} P[M=T^{*}\;\text{where}\;T^{*}\in E(T)] & ={\left(P[\xi_{•}=k]\right)}^{n}\prod_{i=1}^{kn}P[\xi_{\circ }=d_{i}],\\ & ={\left(\frac{1}{C_{k}^{\circ }(\rho_{k,\Omega })}\right)}^{kn}\frac{{\left(B_{k}(\rho_{k,\Omega })\right)}^{n-1}}{\prod_{i=1}^{kn}d_{i}!},\\ & =\frac{{\left(\rho_{k,\Omega }\right)}^{n}}{B_{k}(\rho_{k,\Omega })}\frac{1}{\prod_{i=1}^{kn}d_{i}!}.\; \end{array}$$

For $T^{*}\in E(T)$, let $L(T^{*})$ be the set of different labellings of *T* ^∗^, where all black nodes are labelled with distinct integers (*k* + 1),(*k* + 2),…,(*k* + *n*). Clearly we have $\left|L(T^{*})|=|L(\hat{T})\right|$ for all $T^{*},\hat{T}\in E(T)$. Since the labels on all black nodes of *T* ^∗^ determine the labels on the remaining white nodes, it suffices to count different labellings on black nodes. As we will show next, this leads us to the formula

(12) |L(T∗)|=nn−1d1,d2,⋯,dkn|E(T)|=n!∏i=1kndi!|E(T)|.

First there are *n* ways to label the black root of *T* ^∗^. Second, for the white node with outdegree *d*_1_, there are n−1d1 ways to choose the labels on its black children, where we assume that $T_{1,1},T_{1,2},\cdots ,T_{1,\ell_{1}}$ are distinct subtrees rooted at all these black children appearing with multiplicities $m_{1,1},m_{1,2},\cdots ,m_{1,\ell_{1}}$, respectively. Then $m_{1,1}+m_{1,2}+\cdots +m_{1,\ell_{1}}=d_{1}$ and there are

δd1:=d1m1,1,m1,2,⋯,m1,ℓ1

ways to assign *d*_1_ labels to all these black children. We continue this process for the remaining white nodes with outdegrees *d*_2_,…,*d*_*kn*_ and obtain

|L(T∗)|=nn−1d1n−1−d1d2⋯n−1−∑i=1kn−1didkn∏i=1knδdi=nn−1d1,d2,⋯,dkn∏i=1knδdi,

where $\delta_{d_{i}}$ (2 ≤ *i* ≤ *kn*) is defined in the same way as $\delta_{d_{1}}$. Assume that $T_{i,1},T_{i,2},\cdots ,T_{i,\ell_{i}}$ are distinct subtrees rooted at each of the black children of a white node with outdegree *d*_*i*_, which appear with multiplicities $m_{i,1},m_{i,2},\cdots ,m_{i,\ell_{i}}$, respectively. Then $m_{i,1}+m_{i,2}+\cdots +m_{i,\ell_{i}}=d_{i}$ and there are

δdi:=dimi,1,mi,2,⋯,mi,ℓi

ways to distribute *d*_*i*_ labels on the black children of a white node with outdegree *d*_*i*_. We note that $\prod_{i=1}^{kn}\delta_{d_{i}}$ is exactly the number |E(T)| of different embeddings of *T* into the plane, where only the children of every white node are equipped with the left‐to‐right order. Thus, (12) follows.

For every $T^{*}\in E(T)$ we choose a labelling of *T* ^∗^ uniformly at random, and there is only one labelling of *T* ^∗^ such that *B* is generated after step (3). That is, $P[B_{k}=B|M=T^{*}]={\left(|L(T^{*})|\right)}^{-1}$. Consequently, the probability of drawing *B* of size *n* is given by

$$\begin{array}{ll} P[B_{k}=B]=\sum_{T^{*}\in E(T)}P[M=T^{*}]P[B_{k}=B|M=T^{*}]=P[M=T^{*}]\frac{\left|E(T)\right|}{\left|L(T^{*})\right|}=\frac{{\left(\rho_{k,\Omega }\right)}^{n}}{n!\;B_{k}(\rho_{k,\Omega })}. & \; \end{array}$$

This proves the lemma.

Recall that by (1) every element of the class $C_{k}$ may be decomposed into a collection of elements from the class $B_{k}$, where the number of components belongs to Ω. Let *η*_∘_ denote a random integer from Ω with distribution given by

(13) $$\begin{array}{ll} P[\eta_{\circ }=i]=\frac{1}{C_{k}(\rho_{k,\Omega })}\frac{{\left(B_{k}(\rho_{k,\Omega })\right)}^{i}}{i!}, & \; \end{array}$$

for all *i* ∈ Ω. We are going to make use of the following critical Boltzmann sampling procedure for the class $C_{k}$, which will be used in the proof of Theorem 2; see Section 4. It provides a coupling of a random (*k*,Ω)‐coding tree with a forest of (*ξ*_•_,*ξ*_∘_)‐multitype Galton‐Watson trees. (For the reader's convenience we provide a self‐contained proof, but the result may also be deduced from more general sampling principles given in 22.)

Lemma 4 *The following procedure terminates almost surely:*

1. *Draw η*_∘_ *independent copies* $\left(M_{1},M_{2},\cdots ,M_{\eta_{\circ }}\right)$ *of* (*ξ*_•_,*ξ*_∘_)‐*multitype Galton‐Watson trees*.
2. *Choose a uniform random ordered set partition* $\left(L^{1},L^{2},\cdots ,L^{\eta_{\circ }}\right)$ *of* $\left[\sum_{i=1}^{\eta_{\circ }}|M_{i}|+k]\setminus [k\right]$ *such that* $\left|L^{i}|=|M_{i}\right|$.
3. *For each i, choose a uniform random labelling of* $M_{i}$ *such that all black nodes of* $M_{i}$ *are labelled with distinct integers from* $L^{i}$.
4. *Form a sequence* $\left(B^{1},B^{2},\cdots ,B^{\eta_{\circ }}\right)$ *by adding a white root labelled with the sequence* (1,2,…,*k*) *to every* $M_{i}$. *Glue together the white roots of all* $B^{i}$, 1 ≤ *i* ≤ *η*_∘_.

*This procedure draws a random* ○‐*rooted* (*k*,Ω)‐*coding tree* C_*k*_ *according to a critical Boltzmann distribution. That is, for every* $C\in C_{n,k}$ *(of size n) we have*

$$P[C_{k}=C]=\frac{{\left(\rho_{k,\Omega }\right)}^{n}}{n!\;C_{k}(\rho_{k,\Omega })}.$$

For any ∘‐rooted (*k*,Ω)‐coding tree $C\in C_{n,k}$, assume that *C* can be decomposed into a set {*B* ^1^,*B* ^2^,…,*B* ^*t*^} of $B_{k}$‐objects. If *L* ^*i*^ denotes the label set of *B* ^*i*^ for all 1 ≤ *i* ≤ *t*, then {*L* ^1^,*L* ^2^,…,*L* ^*t*^} forms a partition of the set [*n* + *k*]∖[*k*].

The event *C*_*k*_ = *C* means that *η*_∘_ = *t* and $\left\{B^{1},B^{2},\cdots ,B^{t}\}=\{B^{1},B^{2},\cdots ,B^{t}\right\}$. In other words,

$$\begin{array}{ll} P[C_{k}=C]=P[\eta_{\circ }=t]P[\{B^{1},B^{2},\cdots ,B^{t}\}=\{B^{1},B^{2},\cdots ,B^{t}\}]. & \; \end{array}$$

For any permutation *π* = *π*_1_ *π*_2_⋯*π*_*t*_ ∈ *S*_*t*_ we have

$$P[(B^{1},B^{2},\cdots ,B^{t})=(B^{1},B^{2},\cdots ,B^{t})]=P[(B^{1},B^{2},\cdots ,B^{t})=(B^{\pi_{1}},B^{\pi_{2}},\cdots ,B^{\pi_{t}})],$$

since $B^{i}\overset{d}{=}B_{k}$ (for 1 ≤ *i* ≤ *t*), which leads to

(14) $$\begin{array}{ll} P[C_{k}=C]=t!\;P[\eta_{\circ }=t]\cdot P[(B^{1},B^{2},\cdots ,B^{t})=(B^{1},B^{2},\cdots ,B^{t})]. & \; \end{array}$$

We may express the probability $P[(B^{1},\cdots ,B^{t})=(B^{1},\cdots ,B^{t})]$ by the product

$$\begin{array}{ll} P[(B^{1},\cdots ,B^{t})=(B^{1},\cdots ,B^{t})|(L^{1},\cdots ,L^{t})=(L^{1},\cdots ,L^{t})]\cdot P[(L^{1},\cdots ,L^{t})=(L^{1},\cdots ,L^{t})]. & \; \end{array}$$

It follows from Lemma 3 that $B^{i}\overset{d}{=}B_{k}$ is uniformly distributed among the $\left|B_{|L^{i}|,k}\right|$ many $B_{k}$‐objects with the label set $L^{i}$ for all *i*. Let us set *ℓ*_*i*_ = |*L* ^*i*^| for all 1 ≤ *i* ≤ *t*. Then

(15) $$\begin{array}{ll} P[(B^{1},B^{2},\cdots ,B^{t})=(B^{1},B^{2},\cdots ,B^{t})]=P[(L^{1},L^{2},\cdots ,L^{t})=(L^{1},L^{2},\cdots ,L^{t})]\prod_{i=1}^{t}\frac{1}{\left|B_{\ell_{i},k}\right|}. & \; \end{array}$$

Moreover, in view of Lemma 3, we may write

$$\begin{array}{ll} P[(L^{1},L^{2},\cdots ,L^{t})=(L^{1},L^{2},\cdots ,L^{t})] & =\frac{\ell_{1}!\ell_{2}!\cdots \ell_{t}!}{n!}P[(|L^{1}|,|L^{2}|,\cdots ,|L^{t}|)=(\ell_{1},\ell_{2},\cdots ,\ell_{t})]\;\\ & =\frac{1}{n!}\prod_{i=1}^{t}\frac{|B_{\ell_{i},k}|{\left(\rho_{k,\Omega }\right)}^{\ell_{i}}}{B_{k}(\rho_{k,\Omega })}.\; \end{array}$$

Hence (15) now reads

$$P[(B^{1},B^{2},\cdots ,B^{t})=(B^{1},B^{2},\cdots ,B^{t})]=\frac{{\left(\rho_{k,\Omega }\right)}^{n}}{n!B_{k}{\left(\rho_{k,\Omega }\right)}^{t}}.$$

Combining this with (13) and (14) yields

$$P[C_{k}=C]=\frac{{\left(\rho_{k,\Omega }\right)}^{n}}{n!\;C_{k}(\rho_{k,\Omega })},$$

which completes the proof of the lemma.

Note that ∘ − • (*k*,Ω)‐coding trees satisfy the specification (4), but the graph distance does not represent the distance relation in the corresponding Ω‐*k*‐trees; see Figure 2. Let *B*_*n*,*k*_ denote a random ∘ − • (*k*,Ω)‐coding tree that is uniformly chosen from the class $B_{n,k}$, that is, B_*n*,*k*_ = (B_*k*_:|B_*k*_| = *n*) where B_*k*_ is a random (*k*,Ω)‐coding tree that is generated by Lemma 3.

### 2.4 Ω‐k‐tree distance algorithm

Let *C*_*n*,*k*_ be a random (*k*,Ω)‐coding tree and $G_{n,k}^{□}=\phi^{-1}(C_{n,k})$ be the corresponding Ω‐*k*‐tree under the inverse bijection *𝜑* ^−1^, where *𝜑* is given in section 2.2. In particular $G_{n,k}^{□}$ is rooted at the front [*k*]. We use the notation (*i* ^*m*^,*j* ^*k* − *m*^) to represent a sequence of length *k* with *m* occurrences of *i*, followed by (*k* − *m*) occurrences of *j*.

The purpose of the following procedure is to determine the distances to vertex 1 in an Ω‐*k*‐tree $G_{n,k}^{□}$. More precisely Darrasse and Soria 18 provided an algorithm for this task by marking the distances on the corresponding (*k*,Ω)‐coding tree *C*_*n*,*k*_, which is similar to the algorithm given by Proskurowski in 59. Note that every black node of the (*k*,Ω)‐coding tree is related to a vertex of the corresponding Ω‐*k*‐tree via its label, and the vertices that label a white node of the (*k*,Ω)‐coding tree represent *k* vertices that constitute a front of the corresponding Ω‐*k*‐tree.

Darrasse and Soria's algorithm runs as follows:

Algorithm 1 Distances in an Ω‐*k*‐tree

Input: a (*k*,Ω)‐coding tree *C* and a sequence ${\left(a_{i}\right)}_{i=1}^{k}=(0,1^{k-1})$

Output: an association table (vertex, distance)

$p:=\min{\left\{a_{i}\right\}}_{i=1}^{k}+1$ and *A* = ∅ for all sons *v* of the root *C* do

A:=A∪{(v,p)}

for *i*: = 1→*k* do

*A*←*A*∪ the recursive call on the *i*th son of *v* and (*a*_1_,…,*a*_*i* − 1_,*p*,*a*_*i* + 1_,…,*a*_*k*_)

return *A*

**Figure 4:**
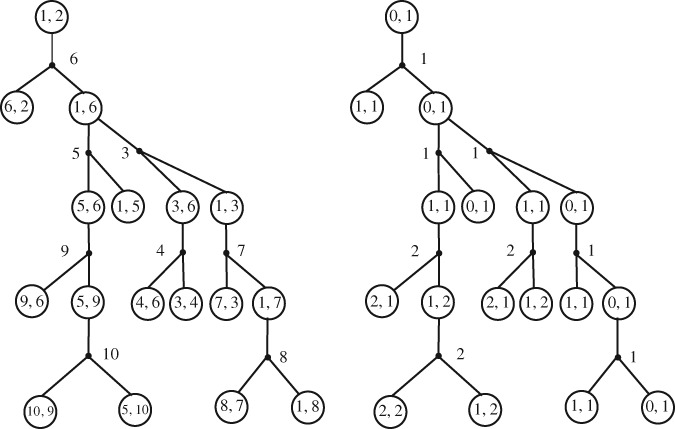
When Ω = {0,1,2,3}, a (2,Ω)‐coding tree (left) and the corresponding distance of every black node to vertex 1 (right)

For example, we implement this algorithm on the (2,Ω)‐coding tree (right) in Figure 2, which provides the distance of every black node to vertex 1 in Figure 4. The distance sequences on the white nodes help us to recursively mark the distances on the black nodes.

Remark 1 Based on this distance algorithm, Darrasse and Soria used a generating function approach to deduce a Rayleigh limiting distribution for the expected distances (scaled by $\sqrt{n}$) between pairs of vertices in a random *k*‐tree; see 18.

### 2.5 Gromov‐Hausdorff‐Prokhorov convergence and the CRT

We shall briefly recall some facts concerning the CRT and the Gromov‐Hausdorff‐Prokhorov metric. We refer the reader to 2, 15, 26 and [55, Sec. 6] for more details. Let *g*:[0,1]→[0,*∞*) be a non‐negative continuous function such that *g*(0) = *g*(1) = 0. We may use it to define a pseudo‐metric ${\tilde{d}}_{g}$ by setting

$$\begin{array}{lcr} {\tilde{d}}_{g}(u,v)=g(u)+g(v)-2\underset{\begin{array}{c} \min\{u,v\}\leq s\leq \max\{u,v\} \end{array}}{\inf}g(s) & & \end{array}$$

for all *u*,*v* ∈ [0,1]. Define an equivalence relation ∼ on [0,1] by letting *x*∼*y* if ${\tilde{d}}_{g}(x,y)=0$. Let *T*_*g*_ = [0,1]/∼ and let *d*_*g*_ be the metric induced on *T*_*g*_ by ${\tilde{d}}_{g}$. Then the associated metric space (*T*_*g*_,*d*_*g*_) is an R‐tree (see 31). We define the *CRT* to be the R‐tree $\left(T_{e},d_{e}\right)$ encoded by the *Brownian excursion of duration onee* = (*e*(*t*),0 ≤ *t* ≤ 1). The random metric space $\left(T_{e},d_{e}\right)$ is endowed with the probability measure *μ*_*e*_ which is the push‐forward of the Lebesgue measure on [0,1].

We consider the CRT as a random point in the set K of all isometry‐equivalence classes of compact measured metric spaces equipped with the *Gromov‐Hausdorff‐Prokhorov distance d*_GHP_. We recall the definition of this metric, starting with the definition of Hausdorff distance.

Given two compact subsets *K*_1_,*K*_2_ of a metric space (*X*,*d*_*X*_), their *Hausdorff distance* is

$$d_{H}(K_{1},K_{2})=\inf\{\epsilon >0|K_{2}\subset {\left(K_{1}\right)}^{\epsilon },K_{1}\subset {\left(K_{2}\right)}^{\epsilon }\}.$$

where $A^{\epsilon }=\{x\in X:\inf_{y\in A}d_{X}(x,y)<\epsilon \}$ is the *ϵ*‐neighborhood of *A*. The *Prokhorov distance* between two Borel probability measures *μ*_1_,*μ*_2_ on *X* is defined by

$$d_{P}(\mu_{1},\mu_{2})=\inf\{\epsilon >0|\;\text{for all closed}A\subset X:\mu_{1}(A)\leq \mu_{2}(A^{\epsilon })+\epsilon ,\mu_{2}(A)\leq \mu_{1}(A^{\epsilon })+\epsilon \}.$$

Let (*X*,*d*_1_,*μ*_1_), (*Y*,*d*_2_,*μ*_2_) be compact metric spaces equipped with Borel probability measures. For any metric space (*E*,*d*_*E*_) and isometric embeddings *ι*_1_:*X*→*E* and *ι*_2_:*Y*→*E* we may consider the push‐forward measures $\mu_{1}\iota_{1}^{-1}$ and $\mu_{2}\iota_{2}^{-1}$ on *E*. The *Gromov‐Hausdorff‐Prokhorov distance* between these two spaces is given by

(16) $$\begin{array}{ll} d_{\text{GHP}}((X,d_{1},\mu_{1}),(Y,d_{2},\mu_{2}))=\underset{\iota_{1},\iota_{2}}{\inf}\max(d_{H}(\iota_{1}(X),\iota_{2}(Y)),d_{P}(\mu_{1}\iota_{1}^{-1},\mu_{2}\iota_{2}^{-1})), & \; \end{array}$$

where the infimum is taken over all isometric embeddings *ι*_1_,*ι*_2_ from *X*,*Y* into any possible common space (*E*,*d*_*E*_).

The Gromov‐Hausdorff‐Prokhorov distance satisfies the axioms of a premetric on the collection of compact metric spaces equipped with Borel probability measures. The corresponding metric on the quotient space K is complete and separable. That is, K is a Polish space. For simplicity we do not distinguish between a measured compact metric space and the corresponding equivalence class.

The main result on which the present work builds is the scaling limit for large Galton‐Watson trees due to Aldous:

Theorem 5 ([5]) *Let T*_*n*_ *be a critical Galton‐Watson tree conditioned on having n vertices, where the offspring distribution has finite nonzero variance σ* ^2^. *Let* $\mu_{T_{n}}$ *denote the uniform measure on the set of vertices of T*_*n*_. *Then, as n tends to infinity, T*_*n*_ *with edges rescaled to length* $\sigma /(2\sqrt{n})$ *converges in distribution to the CRT, i.e.,*

$$\begin{array}{lcr} \left(T_{n},\frac{\sigma }{2\sqrt{n}}{\text{dist}}_{T_{n}},\mu_{T_{n}})\overset{d}{\underset{\;}{\to }}(T_{e},d_{e},\mu_{e}\right) & & \end{array}$$

*with respect to the Gromov‐Hausdorff‐Prokhorov distance*.

Whenever there is no risk of confusion, we will write *λX*, instead of (*X*,*λd*_*X*_,*μ*_*X*_) for any scalar factor *λ* > 0 and any compact metric space (*X*,*d*_*X*_) that is equipped with a Borel probability measure *μ*_*X*_. Hence Theorem 5 may be stated by

$$\frac{\sigma }{2\sqrt{n}}T_{n}\overset{d}{\underset{\;}{\to }}T_{e}.$$

### 2.6 Local convergence

Let X denote the collection of rooted graphs that are connected and locally finite. Given two rooted graphs *G* ^∗^ = (*G*,*v*_*G*_) and *H* ^∗^ = (*H*,*v*_*H*_) from X, we define the distance

$$d(G^{*},H^{*})=2^{-\sup\{m\in N_{0}\;|\;U_{m}(G,v_{G})≃U_{m}(H,v_{H})\}}$$

where *U*_*m*_(*G*,*v*_*G*_) denotes the rooted subgraph of *G* induced by all vertices with graph‐distance at most *m* from the root *v*_*G*_, and *U*_*m*_(*G*,*v*_*G*_)≃*U*_*m*_(*H*,*v*_*H*_) denotes that the two subgraphs are isomorphic as rooted graphs. The distance *d* satisfies the axioms of a premetric and two elements from X have distance zero from each other if and only if they are isomorphic as rooted graphs. Hence *d* defines a complete and separable metric on the collection of all isomorphism classes of graphs from X 15, 26.

A random rooted graph $G_{n}^{*}=(G_{n},v_{n})$ from X converges in the local weak sense towards a random element $G_{\infty }^{*}=(G_{\infty },v_{\infty })$, denoted by

$$(G_{n},v_{n})\overset{d}{\underset{\;}{\to }}(G_{\infty },v_{\infty }),$$

if the corresponding isomorphism classes converge weakly with respect to the metric d. This is equivalent to requiring that for all fixed positive numbers *r*, and for all rooted graphs (*G*,*v*) it holds that

(17) $$\begin{array}{ll} \lim_{n\to \infty }P[U_{r}(G_{n},v_{n})≃(G,v)]=P[U_{r}(G_{\infty },v_{\infty })≃(G,v)]. & \; \end{array}$$

## 3 PROOF OF THEOREM 1

We recall that *G*_*n*,*k*_ and $G_{n,k}^{□}=\phi^{-1}(C_{n,k})$ are identically distributed as random graphs. Hence it suffices to study the latter. We also recall *C*_*n*,*k*_ denotes a random ∘‐rooted (*k*,Ω)‐coding tree of size *n* that is uniformly selected from the class $C_{n,k}$, and that *C*_*n*,*k*_ can be identified with a set of ∘ − • (*k*,Ω)‐coding trees, all of which have the same white root as *C*_*n*,*k*_.

We denote by *L*_*n*,*k*_ one of the largest ∘ − • (*k*,Ω)‐coding trees that is contained in *C*_*n*,*k*_ and denote by *L*_*n*,*k*_ the size of *L*_*n*,*k*_. Equations (2) and (4) allow us to employ a unified analytic framework given by Xavier Gourdon 35. As mentioned in section 2.2 we assume that we are in the aperiodic case, that is, we assume that *z* = *ρ*_*k*,Ω_ is the unique dominant singularity of B_*k*_(*z*). By general theory (see 20, 28) it follows that the singular expansion of B_*k*_(*z*) is given by

$$\begin{array}{ll} B_{k}(z)=g(z)-h(z)\sqrt{1-\frac{z}{\rho_{k,\Omega }}}(1+O(z-\rho_{k,\Omega })) & \; \end{array}$$

where *g*(*z*),*h*(*z*) are analytic around *z* = *ρ*_*k*,Ω_ and *z* may vary in a Δ‐domain of *ρ*_*k*,Ω_

$$\begin{array}{ll} \Delta =\{z\in C,|z|\leq \rho_{k,\Omega }(1+\eta ),|\arg(z-\rho_{k,\Omega })|\geq \varphi \} & \; \end{array}$$

for some *η* > 0 and 0 < *φ* < *π*/2. Furthermore, in view of (2), we can express *C*_*k*_(*z*) = *F*(B_*k*_(*z*)) where $F(w)=\sum_{i\in \Omega }w^{i}{\left(i!\right)}^{-1}$ is an entire function. Thus, *ρ*_*k*,Ω_ is also the dominant singularity of *C*_*k*_(*z*). Hence, we are in the so‐called non‐critical case of 35. By setting *ℓ* = 2, *m* = *n* − *n* ^1/2 − *ϵ*^, *β* = 0 and *α* = 1/2 of Theorem 2 from 35, we have, for every *ϵ* (with 0 < *ϵ* < 1/2) and for some constant *c* > 0

(18) $$\begin{array}{ll} \lim_{n\to \infty }P[L_{n,k}\leq n-n^{\frac{1}{2}-\epsilon }]=\lim_{n\to \infty }(cn^{-\frac{1}{2}}+o(n^{-\frac{1}{2}}))=0. & \; \end{array}$$

This implies that

$$d_{\text{GHP}}\left(\frac{kH_{k}\sigma_{\Omega }}{2\sqrt{n}}\phi^{-1}(C_{n,k}),\frac{kH_{k}\sigma_{\Omega }}{2\sqrt{n}}\phi^{-1}(L_{n,k})\right)\overset{p}{\underset{\;}{\to }}0.$$

Hence in order to prove the Gromov‐Hausdorff‐Prokhorov scaling limit for $G_{n}^{□}=\phi^{-1}(C_{n,k})$ it suffices to prove such a limit for the randomly sized $B_{k}$‐object $\phi^{-1}(L_{n,k})$. We know that *L*_*n*,*k*_ conditioned on having a fixed size is distributed like a uniform $B_{k}$‐object of this size. As the random size *L*_*n*,*k*_ tends weakly towards infinity, it is sufficient to show

(19) $$\begin{array}{ll} \frac{kH_{k}\sigma_{\Omega }}{2\sqrt{n}}G_{n,k}^{•}\overset{d}{\underset{\;}{\to }}T_{e} & \; \end{array}$$

with respect to the Gromov‐Hausdorff‐Prokhorov distance. (Recall that $G_{n,k}^{•}=\phi^{-1}(B_{n,k})$.)

By Lemma 3 we may assume that *B*_*n*,*k*_ = (B_*k*_:|B_*k*_| = *n*). It is clear that any black node has *k* white children and the number of black children *ξ*_∘_ of the white node in *B*_*n*,*k*_, other than the white root, follows the probability distribution (10). This implies that the number of black grandchildren *ξ*_• − •_ of any black node is the sum of *k* independent copies of *ξ*_∘_, that is,

$$\begin{array}{ll} \xi_{•-•}=\xi_{\circ ,1}+\xi_{\circ ,2}+\cdots +\xi_{\circ ,k},\text{where}\;\;\xi_{\circ ,i}\overset{d}{=}\xi_{\circ }. & \; \end{array}$$

So it satisfies

(20) $$\begin{array}{ll} E\xi_{•-•}=kE\xi_{\circ }=1, & \; \end{array}$$

where $E\;\xi_{\circ }=k^{-1}$ is obtained from (6) and (10), namely,

(21) $$\begin{array}{ll} E\;\xi_{\circ } & =\sum_{i\in \Omega }i\;{\left(C_{k}(\rho_{k,\Omega })\right)}^{-1}\;\frac{{\left(B_{k}(\rho_{k,\Omega })\right)}^{i}}{i!}=k^{-1}.\; \end{array}$$

For any two black nodes *x*,*y* in B_*n*,*k*_, we set

(22) $$\begin{array}{ll} d_{B_{n,k}}(x,y)=\frac{1}{2}{\mathrm{dist}}_{B_{n,k}}(x,y). & \; \end{array}$$

For example, consider the (2,Ω)‐coding tree in Figure 2. There we have *n* = 8 and *k* = 2. Let *x*,*y* be the black nodes labelled by 6,9 respectively. Then $d_{B_{8,2}}(x,y)=2$. For the case *k* ≠ 1, the distance $d_{B_{n,k}}(x,y)$ of two black nodes *x*,*y* in B_*n*,*k*_ is different from the distance ${\mathrm{dist}}_{G_{n,k}^{•}}(x,y)$ of *x*,*y* in the original Ω‐*k*‐tree $G_{n,k}^{•}$. In order to represent the distances ${\mathrm{dist}}_{G_{n,k}^{•}}(x,y)$ for any two black nodes *x*,*y* in the tree B_*n*,*k*_, we need to decompose B_*n*,*k*_ into *blocks* according to the distance table from Algorithm 1. We recall that Algorithm 1 marks every black node with a distance and every white node with a distance sequence.

With the help of these labels, we define (so‐called) *i‐blocks* (*i* = 1,2,…) that decompose the random tree B_*n*,*k*_. A *1‐block* of B_*n*,*k*_ is any subtree *T* of B_*n*,*k*_ such that

- *T* is rooted at the white root of B_*n*,*k*_ and *T* is induced by this node and all the black descendants that are at distance one from vertex 1,

while an *i‐block* of B_*n*,*k*_, for *i* ≥ 2, is any subtree *T* of B_*n*,*k*_ such that

- *T* is rooted at a white node with distance sequence ((*i* − 1)^*k*^) and *T* is induced by this node and all its black descendants that are at distance *i* from vertex 1.

**Figure 5:**
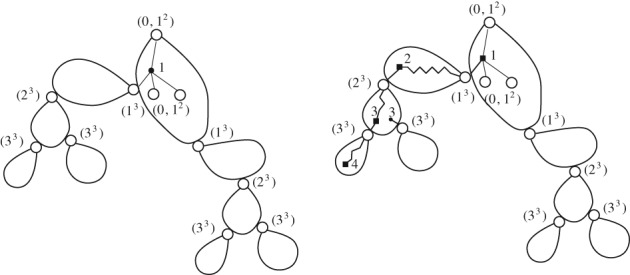
A decomposition of a random (3,Ω)‐coding tree B_n,3_ into blocks (left) where the triple (a,b,c) of integers represents the distance sequence on the root of the block. A spine (right) consists of selected nodes in B_n,3_

Note that there is only one 1‐block for any B_*n*,*k*_, but for *i* ≥ 2, $B_{n,k}$ could contain many *i*‐blocks; see Figure 5. For any two black nodes *x*,*y* in B_*n*,*k*_, let

$$\delta_{B_{n,k}}(x,y)=a-1$$

where *a* is the minimal number of blocks necessary to cover the path connecting *x* and *y*. In particular if *x*,*y* are in the same block of B_*n*,*k*_, then $\delta_{B_{n,k}}(x,y)=0$. The following lemma will show that, for any two black nodes *x*,*y*, the distance ${\mathrm{dist}}_{G_{n,k}^{•}}(x,y)$ is almost the same as the block‐distance $\delta_{B_{n,k}}(x,y)$.

Lemma 6 *Let* B_*k*_ *be the random* ∘ − • (*k*,Ω)‐*coding tree given by Lemma* 3 *and let* B_*n*,*k*_ *denote the tree* B_*k*_ *conditioned on having n black nodes. Furthermore let* $G_{n,k}^{•}=\phi^{-1}(B_{n,k})$ *be the corresponding* Ω‐*k‐tree of* B_*n*,*k*_ *under the inverse bijection 𝜑* ^−1^. *Then for any two black nodes x*,*y of* $G_{n,k}^{•}$,

(23) $$\delta_{B_{n,k}}(x,y)\leq {\mathrm{dist}}_{G_{n,k}^{•}}(x,y)\leq \delta_{B_{n,k}}(x,y)+3.$$

If *x*,*y* are in the same block, i.e., $\delta_{B_{n,k}}(x,y)=0$. If both of them are in a 1‐block, then

$${\mathrm{dist}}_{G_{n,k}^{•}}(x,y)\leq {\mathrm{dist}}_{G_{n,k}^{•}}(x,1)+{\mathrm{dist}}_{G_{n,k}^{•}}(y,1)=2=\delta_{B_{n,k}}(x,y)+2.$$

If both of them are in an (*i* + 1)‐block *T* for some *i* ≥ 1, recall that the root of any (*i* + 1)‐block is a white node with distance sequence (*i* ^*k*^). We assume that the root of *T* has label (*a*_1_,*a*_2_,…,*a*_*k*_). Then for *x* ∈ *T*, there exists an integer *p* such that ${\mathrm{dist}}_{G_{n,k}^{•}}(a_{p},x)=1$. Otherwise if for all 1 ≤ *m* ≤ *k* we have ${\mathrm{dist}}_{G_{n,k}^{•}}(a_{m},x)>1$. It follows that ${\mathrm{dist}}_{G_{n,k}^{•}}(x,1)>i+1$, which contradicts the fact that *x* is contained in an (*i* + 1)‐block. Similarly, there is an integer *q* such that ${\mathrm{dist}}_{G_{n,k}^{•}}(a_{q},y)=1$. Consequently

$${\mathrm{dist}}_{G_{n,k}^{•}}(x,y)\leq {\mathrm{dist}}_{G_{n,k}^{•}}(a_{p},x)+{\mathrm{dist}}_{G_{n,k}^{•}}(a_{q},y)+{\mathrm{dist}}_{G_{n,k}^{•}}(a_{q},a_{p})=3,$$

which implies (23).

If *x*,*y* are not in the same block, let *b* be the last common ancestor of *x* and *y* in B_*n*,*k*_ and we suppose that *b* is a black node because the argument for the case when *b* is a white node follows analogously. Let *a*_1_ (resp. *b*_1_) be the second black node on the path *b* − *ν*_1_ − *a*_1_ − ⋯ − ∘ − *x* (resp. *b* − *ν*_2_ − *b*_1_ − ⋯ − ∘ − *y*) in B_*n*,*k*_. Then one of the shortest paths connecting *x* and *y* in $G_{n,k}^{•}$ must pass node *b*, which implies that

(24) $$\begin{array}{ll} {\mathrm{dist}}_{G_{n,k}^{•}}(x,y)={\mathrm{dist}}_{G_{n,k}^{•}}(x,b)+{\mathrm{dist}}_{G_{n,k}^{•}}(y,b). & \; \end{array}$$

This is true because the Ω‐*k*‐tree corresponding to the subtree of B_*n*,*k*_ rooted at *a*_1_ and the Ω‐*k*‐tree corresponding to the subtree of B_*n*,*k*_ rooted at *b*_1_ are completely disjoint if we remove all (*k* − 1) common vertices of *ν*_1_ and *ν*_2_ from $G_{n,k}^{•}$. These (*k* − 1) common vertices form a (*k* − 1)‐clique and one of these common vertices is *b*. We shall show that $\delta_{B_{n,k}}(x,y)\leq {\mathrm{dist}}_{G_{n,k}^{•}}(x,y)$. Note that

$$\delta_{B_{n,k}}(x,b)={\mathrm{dist}}_{G_{n,k}^{•}}(x,1)-{\mathrm{dist}}_{G_{n,k}^{•}}(b,1)\leq {\mathrm{dist}}_{G_{n,k}^{•}}(x,b)$$

and in the same way $\delta_{B_{n,k}}(y,b)\leq {\mathrm{dist}}_{G_{n,k}^{•}}(y,b)$, which, together with (24), implies that

$$\delta_{B_{n,k}}(x,y)=\delta_{B_{n,k}}(x,b)+\delta_{B_{n,k}}(y,b)\leq {\mathrm{dist}}_{G_{n,k}^{•}}(x,b)+{\mathrm{dist}}_{G_{n,k}^{•}}(y,b)={\mathrm{dist}}_{G_{n,k}^{•}}(x,y).$$

If *x* is contained in an (*i* + 1)‐block, then there must exist a black node *v*_1_ on the path *b* − ∘ − *a*_1_ − ⋯ − ∘ − *x*, such that ${\mathrm{dist}}_{G_{n,k}^{•}}(x,v_{1})=1$ and *v*_1_ is contained in an *i*‐block. For the node *v*_1_, there exists a black node *v*_2_ on the path such that *v*_2_ is contained in an (*i* − 1)‐block and ${\mathrm{dist}}_{G_{n,k}^{•}}(x,v_{2})=2$. We continue this process until we reach a black node *v*_*t*_ such that *v*_*t*_ and *b* are in the same block and ${\mathrm{dist}}_{G_{n,k}^{•}}(x,v_{t})=t=\delta_{B_{n,k}}(x,b)$. Similarly, we can find a sequence of black nodes *w*_1_,…,*w*_*s*_ from different blocks such that *w*_*s*_ and *b* are in the same block and ${\mathrm{dist}}_{G_{n,k}^{•}}(y,w_{s})=s=\delta_{B_{n,k}}(y,b)$. It follows that

$$\begin{array}{ll} \delta_{B_{n,k}}(x,y)\leq {\mathrm{dist}}_{G_{n,k}^{•}}(x,y) & \leq {\mathrm{dist}}_{G_{n,k}^{•}}(x,v_{t})+{\mathrm{dist}}_{G_{n,k}^{•}}(y,w_{s})+{\mathrm{dist}}_{G_{n,k}^{•}}(v_{t},w_{s})\;\\ & =\delta_{B_{n,k}}(x,b)+\delta_{B_{n,k}}(y,b)+{\mathrm{dist}}_{G_{n,k}^{•}}(v_{t},w_{s})\;\\ & =\delta_{B_{n,k}}(x,y)+{\mathrm{dist}}_{G_{n,k}^{•}}(v_{t},w_{s}).\; \end{array}$$

Since *v*_*t*_ and *w*_*s*_ are in the same block, we have shown that ${\mathrm{dist}}_{G_{n,k}^{•}}(v_{t},w_{s})\leq 3$, as desired.

Lemma 6 allows us to transfer the distance ${\mathrm{dist}}_{G_{n,k}^{•}}(x,y)$ of two vertices *x*,*y* in a random Ω‐*k*‐tree $G_{n,k}^{•}$ to the distance $\delta_{B_{n,k}}(x,y)$ of two blocks in a random tree B_*n*,*k*_. In order to prove the convergence of $G_{n,k}^{•}$ to the CRT $T_{e}$, it is, thus, sufficient to prove that with high probability the difference between $m_{k}\delta_{B_{n,k}}(x,y)$ and $d_{B_{n,k}}(x,y)$ is uniformly small for all choices of *x*,*y*, where $m_{k}$ is a properly chosen constant. For this purpose we consider the *spine* of a size‐biased (*ξ*_•_,*ξ*_∘_)‐multitype Galton‐Watson tree. This construction is adapted from 1 and is a truncated version of the infinite size‐biased Galton‐Watson tree introduced by Kesten 47, Lyons and coworkers 52. Furthermore, this idea has been used in studying the scaling limit of random graphs from subcritical graph classes 57 and was further generalized to the random R‐enriched trees 62.

Let ${\hat{\xi }}_{\circ }$ be a random variable with the *size‐biased* distribution

(25) $$P[{\hat{\xi }}_{\circ }=q]=kq\;P[\xi_{\circ }=q].$$

Note that this is a probability distribution on $N^{+}=\{1,2,\cdots \}$ because ${\hat{\xi }}_{\circ }\geq 1$ and $E\;\xi_{\circ }=k^{-1}$.

The *size‐biased* (*ξ*_•_,*ξ*_∘_)‐*multitype Galton‐Watson tree* ${\hat{M}}^{\left(m\right)}$, where *m* ≥ 1, is now defined as follows. It starts with a *black‐mutant noder* which is a black node and it has white‐node offspring according to *ξ*_•_; see (11). We now choose one of these white‐node children uniformly at random and call it *white‐mutant*. Every other white‐node offspring is called *white‐normal*. Each white‐mutant node has black‐node offspring according to ${\hat{\xi }}_{\circ }$, while each white‐normal node has black‐node offspring according to *ξ*_∘_ (see (10) and all these black‐node offspring are called *black‐normal*. We again choose one of the black‐node children of the white‐mutant node uniformly at random and call it *heir*. All other black‐node children are also called *black‐normal*, where all black‐normal nodes have white‐normal children according to independent copies of *ξ*_•_. The heir is black‐mutant if it has depth less than 2*m*, and we proceed recursively as long as the heir is black‐mutant. Finally if the heir has depth 2*m*, then all black nodes at this level are normal, which again have white‐normal offspring according to independent copies of *ξ*_•_.

Let *h*_*m*_ denote the heir that is the (*m* + 1)th black‐mutant node of ${\hat{M}}^{\left(m\right)}$, then the path from the black root *r* to *h*_*m*_ is called *a spine* of ${\hat{M}}^{\left(m\right)}$. For simplicity, we adopt the notations of $B,T,T^{*},E(T),L(T^{*})$ and outdegree sequence (*d*_1_,*d*_2_,…,*d*_*kn*_) of *T* from the proof of Lemma 3. Our aim is to show that for every $T^{*}\in E(T)$ and every fixed spine *γ* of length 2*m* that connects the root and *v* of *T* ^∗^ we have

(26) $$\begin{array}{ll} P[{\hat{M}}^{\left(m\right)}=T^{*},h_{m}=v]=P[M=T^{*}]. & \; \end{array}$$

The probability that a given black‐mutant node has one white‐mutant child and this white‐mutant child has *q* black‐node children where one of them is chosen as heir is, ${\left(kq\right)}^{-1}P[{\hat{\xi }}_{\circ }=q]=P[\xi_{\circ }=q]$. Hence,

$$\begin{array}{ll} P[{\hat{M}}^{\left(m\right)}=T^{*},h_{m}=v]={\left(P[\xi_{•}=k]\right)}^{n}\prod_{i=1}^{kn}P[\xi_{\circ }=d_{i}]=P[M=T^{*}], & \; \end{array}$$

which implies (26). We recall that B_*k*_ denotes a random (*k*,Ω)‐coding tree that is generated in Lemma 3. Our next claim is

(27) $$P[{\hat{B}}_{k}^{\left(m\right)}=B,h_{m}=v]=P[B_{k}=B],$$

where ${\hat{B}}_{k}^{\left(m\right)}$ is a *size‐biased (k,Ω)‐coding tree* that is constructed as follows:

1. Draw a sized‐biased (*ξ*_•_,*ξ*_∘_)‐multitype Galton‐Watson tree ${\hat{M}}^{\left(m\right)}$.
2. Choose a uniform random labelling of ${\hat{M}}^{\left(m\right)}$ such that all black nodes of ${\hat{M}}^{\left(m\right)}$ are labelled with distinct integers $\left(k+1),(k+2),\cdots ,(k+|{\hat{M}}^{\left(m\right)}|\right)$.
3. Add a white root to ${\hat{M}}^{\left(m\right)}$ and label it with (1,2,…,*k*).

This construction is analogous to the one in Lemma 3, which, in combination with (26), implies that

$$\begin{array}{ll} P[{\hat{B}}_{k}^{\left(m\right)}=B,h_{m}=v]=\frac{E(T)}{\left|L(T^{*})\right|}P[{\hat{M}}^{\left(m\right)}=T^{*},h_{m}=v]=\frac{E(T)}{\left|L(T^{*})\right|}P[M=T^{*}]=P[B_{k}=B]. & \; \end{array}$$

**Figure 6:**
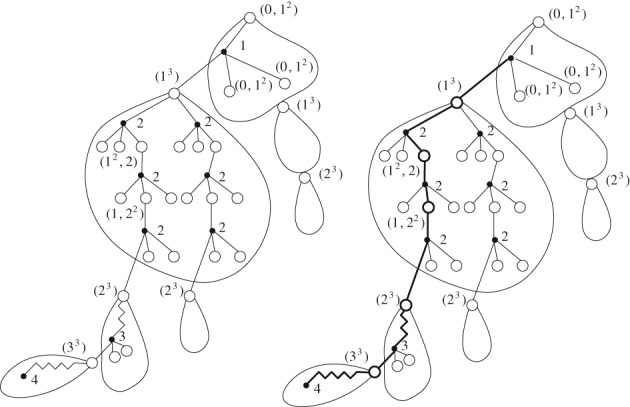
A (3,Ω)‐coding tree $B_{3}$ (left) and a size‐biased one ${\hat{B}}_{3}^{\left(m\right)}$ with a spine γ consisting of all black‐mutant nodes and white‐mutant nodes (right), where every black node is marked with a distance and every white node is marked with a distance sequence

That is, (27) is true. This relation shows, once the spine *γ* is fixed, that the probability that the size‐biased tree ${\hat{B}}_{k}^{\left(m\right)}$ equals *B* is the same as the probability of the event $B_{k}=B$ (see equation (3.2) from 1 for a size‐biased Galton‐Watson tree and Figure 6 for an example).

Lemma 7 *Let* $B_{n,k}$ *be the class of* ∘ − • (*k*,Ω)‐*coding trees of size n such that the white root has label* (1,2,…,*k*) *and let* $B_{n,k}\in B_{n,k}$ *be selected uniformly at random. Let* $m_{k}=kH_{k}$ *and let r be the only black‐node child of the white root of* $B_{n,k}$. *Then for all* 0 < *s*,*ϵ* < 1/2 *and for every black node v* ∈ B_*n*,*k*_ *such that* $d_{B_{n,k}}(r,v)\geq n^{s}$, *the property*

(28) $$\left|d_{B_{n,k}}(r,v)-m_{k}\delta_{B_{n,k}}(r,v)|\leq \epsilon \;d_{B_{n,k}}(r,v\right)$$

*holds with probability tending to 1 as n tends to infinity*.

Suppose that the opposite of (28) is true, i.e., there exists $v^{*}\in B_{k}$ such that

$$\begin{array}{ll} d_{B_{k}}(r,v^{*})\geq |B_{k}{|}^{s}\;\text{and}\;|d_{B_{k}}(r,v^{*})-m_{k}\delta_{B_{k}}(r,v^{*})|>\epsilon \;d_{B_{k}}(r,v^{*}). & \; \end{array}$$

For any such $B_{k}$, fix one black node $v^{*}\in B_{k}$ having this property. Furthermore let $F_{1}$ be the collection of all such pairs $\left(B_{k},v^{*}\right)$. We are going to show that

$$\begin{array}{ll} P[(B_{k},v^{*})\in F_{1}|\;|B_{k}|=n]\to 0,\text{as}\;n\to \infty . & \; \end{array}$$

With the help of Lemma 3 and by applying [20, Theorem 2.19]) we obtain, with $\sigma_{\Omega }^{2}=kV\text{ar}\;\xi_{\circ }$, the asymptotic relation

(29) $$\begin{array}{ll} P[|B_{k}|=n]=\frac{b_{k,\Omega }(n)\rho_{k,\Omega }^{n}}{n!\;B_{k}(\rho_{k,\Omega })}∼\frac{n^{-3/2}}{\sigma_{\Omega }\sqrt{2\pi }}\;\text{as}\;n\to \infty . & \; \end{array}$$

Consequently, we have

(30) $$\begin{array}{ll} P[(B_{k},v^{*})\in F_{1}|\;|B_{k}|=n] & ={\left(P[|B_{k}|=n]\right)}^{-1}P[(B_{k},v^{*})\in F_{1}\;\text{and}\;|B_{k}|=n],\\ & =(\sigma_{\Omega }\sqrt{2\pi })n^{3/2}P[(B_{k},v^{*})\in F_{1}\;\text{and}\;|B_{k}|=n].\; \end{array}$$

Furthermore,

(31) $$\begin{array}{ll} P[(B_{k},v^{*})\in F_{1}\;\text{and}\;|B_{k}|=n]\leq \sum_{n^{s}\leq m\leq n}\sum_{\begin{array}{c} (B,v)\in F_{1}\\ d_{B}(r,v)=m \end{array}}P[B_{n,k}=B]. & \; \end{array}$$

Now for any $(B,v)\in F_{1}$ we consider the spine of *B* that ends at *v*. Then from (27) we see that

(32) $$\begin{array}{ll} \sum_{\begin{array}{c} (B,v)\in F_{1}\\ d_{B}(r,v)=m \end{array}}P[B_{n,k}=B]=\sum_{\begin{array}{c} (B,v)\in F_{1}\\ |B|=n \end{array}}P[{\hat{B}}_{k}^{\left(m\right)}=B,h_{m}=v]. & \; \end{array}$$

Let *r*,*h*_1_,*h*_2_,…,*h*_*m* − 1_,*h*_*m*_ be the black‐mutant nodes contained in the spine of ${\hat{B}}_{k}^{\left(m\right)}$, and let $\delta_{i}=\delta_{{\hat{B}}_{k}^{\left(m\right)}}(r,h_{i})$ be the block‐distance of every *h*_*i*_ to *r*. It follows from (30), (31) and (32) that

(33) $$\begin{array}{ll} P[(B_{k},v^{*})\in F_{1}|\;|B_{k}|=n]\leq (\sigma_{\Omega }\sqrt{2\pi })n^{3/2}\sum_{n^{s}\leq m\leq n}p_{m}, & \; \end{array}$$

where *p*_*m*_ denotes the probability, that the block‐distance *δ*_*m*_ of *h*_*m*_ to *r* in the random size‐biased (*k*,Ω)‐coding tree ${\hat{B}}_{k}^{\left(m\right)}$ satisfies $|m_{k}\delta_{m}-m|>\epsilon m$.

Set *ϑ*_*i*_ = *δ*_*i*_ + 1, where *ϑ*_*i*_ equals the graph‐distance of *h*_*i*_ to vertex 1 in the corresponding Ω‐*k*‐tree $\phi^{-1}({\hat{B}}_{k}^{\left(m\right)})$. In other words, after we implement Algorithm 1 on ${\hat{B}}_{k}^{\left(m\right)}$, the distance marked on *h*_*i*_ is equal to *ϑ*_*i*_.

From Algorithm 1 we observe that *ϑ*_*i* + 1_ only depends on *ϑ*_*i*_ and the distance sequence of the white node that has *h*_*i*_ as a child. Let *X*_*i*_ ≥ 1 be the number of integers contained in this distance sequence that equal *ϑ*_*i*_ − 1, as a result, (*X*_*i*_,*ϑ*_*i*_)_*i*_ is a Markov chain that starts at (*X*_0_,*ϑ*_0_) = (1,1) where *X*_0_ = 1 counts the number of zeros in the distance sequence of the white root (0,1^*k* − 1^) and *ϑ*_0_ = 1 is the distance marked on *r* after using Algorithm 1. Here, for all *i* ≥ 1,

$$\begin{array}{ll} \left((X_{i},\vartheta_{i})||X_{i-1}=x,\vartheta_{i-1}=d\right) & \; \end{array}$$

is distributed as follows.

- We consider a Bernoulli random variable *τ* with $P[\tau =1]=X_{i-1}/k$ and $P[\tau =0]=1-(X_{i-1}/k)$. That is, we make a Bernoulli (*X*_*i* − 1_/*k*) choice.
- If *τ* = 1 and *x* ≥ 2 we set (*X*_*i*_,*ϑ*_*i*_) = (*x* − 1,*d*), if *τ* = 1 and *x* = 1 we set (*X*_*i*_,*ϑ*_*i*_) = (*k*,*d* + 1), and if *τ* = 0 we set (*X*_*i*_,*ϑ*_*i*_) = (*x*,*d*).

In particular, (*X*_*i*_)_*i*_ is a Markov chain with state space {1,2,…,*k*} and transition matrix

$$P=\left(\begin{array}{cccccc} \frac{k-1}{k} & 0 & 0 & \cdots & 0 & \frac{1}{k}\\ \frac{2}{k} & \frac{k-2}{k} & 0 & \cdots & 0 & 0\\ 0 & \frac{3}{k} & \frac{k-3}{k} & \cdots & 0 & 0\\ 0 & 0 & \ddots & \ddots & 0 & 0\\ 0 & 0 & \cdots & \frac{k-1}{k} & \frac{1}{k} & 0\\ 0 & 0 & \cdots & 0 & 1 & 0 \end{array}\right).$$

The stationary distribution $\pi ={\left(\pi_{i}\right)}_{i=1}^{k}$ satisfies *π* = *πP*, which yields

$$\begin{array}{ll} \pi ={\left(\pi_{i}\right)}_{i=1}^{k}=\frac{1}{H_{k}}(1,\frac{1}{2},\cdots ,\frac{1}{k}). & \; \end{array}$$

Since (*X*_*m*_,*ϑ*_*m*_) is a Markov‐additive process, we apply a general large deviation result for these processes [51, Thm. 3.3] and obtain that for every *ϵ* ^∗^ > 0, there is a constant *c*(*ϵ* ^∗^) > 0 such that

$$\begin{array}{ll} P[|m^{-1}(\delta_{m}+1)-\mu |>\epsilon^{*}]\leq \exp(-c(\epsilon^{*})m) & \; \end{array}$$

holds for all *m*, *n* ^*s*^ ≤ *m* ≤ *n*, where $\mu =\pi_{1}/k={\left(kH_{k}\right)}^{-1}=m_{k}^{-1}$ is the mean of a typical step of the chain in the stationary state. This implies that *p*_*m*_ is exponentially small. Hence, from (33) we conclude that $P[(B_{k},v^{*})\in F_{1}|\;|B_{k}|=n]\to 0$ as *n*→*∞*, which completes the proof of the lemma.

Now we are ready to prove our first main result.

Proof of Theorem 1 We recall that the number of black grandchildren *ξ*_• − •_ of any black node in B_*n*,*k*_ satisfies $E\;\xi_{•-•}=kE\;\xi_{\circ }=1$; see (20). Furthermore, *Varξ*_• − •_ = *kVarξ*_∘_. From (10) it follows immediately that 0 < Var *ξ*_∘_ < *∞*, we thus have 0 < Var *ξ*_• − •_ < *∞*.

Let *S*_*n*_ be a critical Galton‐Watson tree conditioned on having size *n*, where the number of offspring follows the distribution of the random variable *ξ*_• − •_. Then by applying Theorem 5 we obtain

$$\begin{array}{ll} (S_{n},\frac{\sigma_{\Omega }}{2\sqrt{n}}\;{\text{dist}}_{S_{n}},\mu_{S_{n}})\overset{d}{\underset{\;}{\to }}(T_{e},d_{e},\mu_{e})\;\;\text{in the metric space}\;(K,d_{\text{GHP}}), & \; \end{array}$$

where $0<\sigma_{\Omega }^{2}=Var\;\xi_{•-•}<\infty$. Since $d_{\text{GHP}}((S_{n},{\text{dist}}_{S_{n}},\mu_{S_{n}}),(B_{n,k},d_{B_{n,k}},\mu_{B_{n,k}}))=0$, we have

(34) $$(B_{n,k},\frac{\sigma_{\Omega }}{2\sqrt{n}}\;d_{B_{n,k}},\mu_{B_{n,k}})\overset{d}{\underset{\;}{\to }}(T_{e},d_{e},\mu_{e})\;\;\text{in the metric space}\;(K,d_{\text{GHP}}).$$

In order to prove Theorem 1, it suffices to show (19), as we have argued in section 2.1 and Section 3. In view of $\phi (G_{n,k}^{•})=B_{n,k}$, Lemma 6 and (34), it is also sufficient to prove

(35) $$\begin{array}{ll} d_{\text{GHP}}((B_{n,k},\frac{d_{B_{n,k}}}{\sqrt{n}},\mu_{B_{n,k}}),(B_{n,k},\frac{m_{k}\delta_{B_{n,k}}}{\sqrt{n}},\mu_{B_{n,k}}))\overset{p}{\underset{\;}{\to }}0. & \; \end{array}$$

Let *r* be the only black‐node child of the white root of $B_{n,k}$, then for any two black nodes *u*,*v* of $B_{n,k}$, let *o* be the last common ancestor of *u*,*v*. Without loss of generality, suppose that *o* is a black node, then one can easily check that

$$\begin{array}{ll} |\delta_{B_{n,k}}(u,v)-(\delta_{B_{n,k}}(r,u)+\delta_{B_{n,k}}(r,v)-2\delta_{B_{n,k}}(r,o))|=0 & \; \end{array}$$

which is also true if $\delta_{B_{n,k}}$ is replaced by $d_{B_{n,k}}$. Thus, in order to show (35), we only need to prove

(36) $$\begin{array}{ll} \frac{1}{\sqrt{n}}\underset{v\in B_{n,k}}{\sup}|d_{B_{n,k}}(r,v)-m_{k}\delta_{B_{n,k}}(r,v)|\overset{p}{\underset{\;}{\to }}0. & \; \end{array}$$

Since $\delta_{B_{n,k}}(r,v)\leq d_{B_{n,k}}(r,v)$, we find that (36) is true for all $v\in B_{n,k}$ such that $d_{B_{n,k}}(r,v)<n^{s}$ for 0 < *s* < 1/2. So we only need to consider vertices $v\in B_{n,k}$ such that $d_{B_{n,k}}(r,v)\geq n^{s}$.

It follows from Lemma 7 that

$$\begin{array}{ll} \frac{1}{\sqrt{n}}\underset{v\in B_{n,k}}{\sup}|d_{B_{n,k}}(r,v)-m_{k}\delta_{B_{n,k}}(r,v)|\leq \frac{\epsilon \;d_{B_{n,k}}(r,v)}{\sqrt{n}}=\frac{\epsilon \;{\text{dist}}_{S_{n}}(r,v)}{\sqrt{n}}\leq \frac{\epsilon \;H(S_{n})}{\sqrt{n}} & \; \end{array}$$

holds with probability tending to 1 as *n* tends to infinity, where $H(S_{n})$ is the height of $S_{n}$. By applying the tail‐bounds and the left‐tail upper bounds for the height $H(S_{n})$ of $S_{n}$ (see 1), we conclude that $\epsilon \;H(S_{n})/\sqrt{n}$ tends to zero as *n* tends to infinity. In consequence, (36) is true. This finally completes the proof of Theorem 1. □

## 4 PROOF OF THEOREM 2

In this section, we are going to construct an infinite Ω‐*k*‐tree G_*∞*,*k*_ that is rooted at a front of distinguishable vertices. We then establish the convergence of $G_{n,k}^{\circ }$ toward this random graph in the sense, that for each fixed integer *ℓ* ≥ 0 the front‐rooted sub‐Ω‐*k*‐tree $U_{\ell }(G_{n,k}^{\circ })$ that is induced by all vertices at distance at most *ℓ* from the marked front, converges in distribution to the corresponding sub‐Ω‐*k*‐tree *U*_*ℓ*_(G_*∞*,*k*_) of the limiting object.

By the discussion in section 2.1, the random Ω‐*k*‐tree $G_{n,k}^{\circ }$ is up to relabelling distributed like the Ω‐*k*‐tree $G_{n,k}^{□}$ that is rooted at a fixed front with labels from 1 to *k*. Hence we only need to study the neighborhoods of the root‐front. If we distinguish any fixed vertex of the marked front in $G_{n,k}^{□}$, for example the vertex with label 1, and also distinguish a fixed vertex of the marked front in G_*∞*,*k*_, then our limit may be interpreted as a classical local weak limit of a sequence of vertex‐rooted random graphs as discussed in section 2.6. This may be justified by the following two arguments. First, as rooted graphs, all *k* possible vertex‐rootings of $G_{n,k}^{□}$ are identically distributed, and we shall see below that the same is true for the limit G_*∞*,*k*_. Second, the *ℓ*‐neighborhood of a vertex of any front‐rooted Ω‐*k*‐tree is always a subgraph of the *ℓ*‐neighborhood of the marked front, and hence weak convergence of the neighborhoods of the front implies weak convergence of the neighborhoods of the vertices.

The strategy of the proof is as follows. We generate a random Ω‐*k*‐tree $G_{n,k}^{□}$ by applying the bijection $\phi^{-1}:C_{n,k}\to G_{n,k}^{□}$ to the random (*k*,Ω)‐coding tree $C_{n,k}$. This random coding tree may be generated by conditioning *C*_*k*_ on having *n* black nodes (as done in Lemma 4). We observe that any ordered tree of white vertices where the outdegree of every vertex is a multiple of *k* may be interpreted as a (*k*,Ω)‐coding tree by adding black vertices in a canonical way. Here different plane trees correspond to the same unlabelled (*k*,Ω)‐tree, but this will not be an issue. We use this construction in order to formulate a coupling of the random (*k*,Ω)‐coding tree *C*_*k*_ with a Galton‐Watson tree T_∘_ that has a modified root‐degree. If we condition this locally modified Galton‐Watson tree on having (*kn* + 1) vertices, then the result *T*_*n*,∘_ corresponds, up to relabelling, to the (*k*,Ω)‐coding tree $C_{n,k}$. By the same kind of arguments that provide local convergence of simply generated trees, we obtain that the random tree *T*_*n*,∘_ converges weakly toward an infinite plane tree T_*∞*,∘_, which may be interpreted as a (*k*,Ω)‐coding tree *C*_*∞*,*k*_, and consequently also as a front‐rooted Ω‐*k*‐tree G_*∞*,*k*_. The final step in the proof is to deduce local convergence of a random Ω‐*k*‐tree $G_{n,k}^{□}$ from this convergence of random trees.

**Figure 7:**
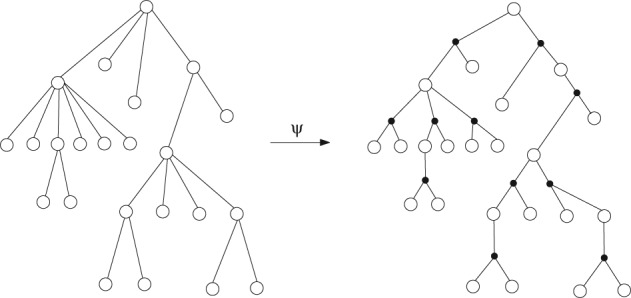
Construction of (k,Ω)‐coding trees out of plane trees where the outdegree of each vertex is a multiple of k, illustrated for the special case k = 2

The construction of a (*k*,Ω)‐coding tree *ψ*(*T*) out of a plane tree *T*, where the outdegree of each vertex is a multiple of *k*, is straight‐forward. We canonically partition the offspring set of each vertex *v* of *T* into an ordered list of groups *G*_1_(*v*),*G*_2_(*v*),… of *k* consecutive vertices. The edges between *v* and its offspring are then deleted, and for each group *G*_*i*_(*v*), we add a black offspring vertex *u*_*i*_(*v*) to *v* and add further edges such that *G*_*i*_(*v*) is the offspring set of *u*_*i*_(*v*). This construction is illustrated in Figure 7.

We may now use this to formulate a coupling of a critical Boltzmann distributed (*k*,Ω)‐coding tree with a modified Galton‐Watson tree. Recall that in Lemma 4 we constructed a random (*k*,Ω)‐coding tree $C_{k}$ by starting with a white root, and connecting it with the roots of a random number *η*_∘_ of independent ∘ − • (*k*,Ω)‐coding trees, where each of them is sampled independently according to the random tree M. Recall that the tree M starts with a black node with *k* white nodes as offspring. Each of the white nodes receives a random number *ξ*_∘_ of black nodes, which follows the distribution given in (10). Then the sampler is applied recursively. That is, every black node receives *k* white nodes as offspring and each of them receives a random number of black offspring, and so on.

Let T_∘_ denote a modified Galton‐Watson tree, where each vertex receives offspring according to an independent copy of *ξ*: = *kξ*_∘_, except for the root, which receives offspring according to *η*: = *kη*_∘_. The order in which the recursion takes place in M does not matter, hence the (*k*,Ω)‐coding tree *ψ*(T_∘_) is up to relabelling distributed like a (*k*,Ω)‐coding tree $C_{k}$. Moreover, let *T*_*n*,∘_ denote the tree T_∘_ conditioned on having (*kn* + 1) vertices, then *ψ*(*T*_*n*,∘_) is distributed as the random (*k*,Ω)‐coding tree C_*n*,*k*_.

Note that (21) implies that Eξ=1, and both *ξ* and *η* have finite exponential moments. We define the size‐biased versions of these offspring distributions by

$$P[\hat{\xi }=i]=iP[\xi =i]\;\text{and}\;P[\hat{\eta }=i]=iP[\eta =i]/E\eta .$$

Let T_*∞*,∘_ denote the following random infinite (but locally finite) plane tree. There are two types of non‐root vertices, mutant and normal. The root receives offspring according to $\hat{\eta }$, and one of its sons is selected uniformly at random and declared mutant, whereas the others are normal. Normal vertices receive offspring according to an independent copy of *ξ*, all of which are normal. Mutant vertices receive offspring according to an independent copy of $\hat{\xi }$, and among them one is selected uniformly at random and declared mutant, whereas the others are normal. Hence T_*∞*,∘_ is an infinite plane tree with a distinguished path that starts at the root and traverses the mutant vertices. We call this path the spine of T_*∞*,∘_.

We describe the convergence of the random tree *T*_*n*,∘_ toward the limit tree T_*∞*,∘_ using a slight modification of the arguments in Janson's survey 42. For each plane tree *T* and each integer *h* ≥ 0 let *T* ^[*h*]^ denote the tree obtained by cutting away all vertices at height larger than *h*.

Lemma 8 *For every integer h* ≥ 0, *it holds that* $T_{n,\circ }^{\left[h\right]}\overset{d}{\underset{\;}{\to }}T_{\infty ,\circ }^{\left[h\right]}.$

It suffices to show, for each plane tree *T* at height *h*, that

(37) $$\begin{array}{ll} \lim_{n\to \infty }P[T_{n,\circ }^{\left[h\right]}=T]=P[T_{\infty ,\circ }^{\left[h\right]}=T]. & \; \end{array}$$

As T_∞,∘_ has infinite height, this already implies that H(*T*_n,∘_) ≥ *h* occurs with probability tending to 1, and consequently $T_{n,\circ }^{\left[h\right]}\overset{d}{\underset{}{\to }}T_{\infty ,\circ }^{\left[h\right]}$. In order to check (37), let *d*_1_,…,*d*_*t*_ denote the depth‐first‐search ordered list of the outdegrees of all vertices in the pruned tree *T* ^[*h* − 1]^. Moreover, let ${\left(\xi_{i}\right)}_{i\in N}$ denote a family of independent copies of *ξ*. Set *N* = *kn* + 1 and *D* = *d*_1_ + ⋯ + *d*_*t*_. The probability $P[|T_{\circ }|=N,T_{\circ }^{\left[h\right]}=T]$ is given by

(38) $$\begin{array}{ll} P[\eta =d_{1}]\cdot \prod_{j=2}^{t}P[\xi =d_{j}]\cdot P\left[D+\sum_{j=t+1}^{N}\xi_{j}=N-1,D+\sum_{j=t+1}^{m}\xi_{j}\geq m\;\text{for all}t<m<N\right]. & \; \end{array}$$

A classical combinatorial observation, also called the cycle lemma, states that for any sequence *x*_1_,…,*x*_*s*_≥ − 1 of integers satisfying $\sum_{i=1}^{s}x_{i}=-r$ for some *r* ≥ 1, there are precisely *r* integers 1 ≤ *u* ≤ *s* such that the cyclically shifted sequence $x_{i}^{\left(u\right)}=x_{1+(i+u)\;\mathrm{mod}\;s}$ satisfies $\sum_{i=1}^{\ell }x_{i}^{\left(u\right)}>r$ for all 1 ≤ *ℓ* ≤ *s* − 1; see for example[42, Lem. 15.3]. Consequently, (38) may be simplified to

(39) $$\begin{array}{ll} \frac{D-t+1}{N-t}\cdot P[\eta =d_{1}]\prod_{j=2}^{t}P[\xi =d_{j}]\cdot P\left[D+\sum_{j=t+1}^{N}\xi_{j}=N-1\right]. & \; \end{array}$$

The tree *T* has precisely (*D* − *t* + 1) vertices at height *h*. Hence the event $T_{\infty ,\circ }^{\left[h\right]}=T$ corresponds to precisely (*D* − *t* + 1) possible outcomes for the first (*h* + 1) levels of *T*_*∞*,∘_, depending on the location for the unique spine vertex at height *h*. Each outcome has the same probability

$${\left(E\eta \right)}^{-1}\cdot P[\eta =d_{1}]\cdot \prod_{j=2}^{t}P[\xi =d_{j}].$$

Thus, $P[T_{\infty ,\circ }^{\left[h\right]}=T]=(D-t+1){\left(E\eta \right)}^{-1}P[\eta =d_{1}]\prod_{j=2}^{t}P[\xi =d_{j}]$ and (38) is simplified to

$$P[|T_{\circ }|=N,T_{\circ }^{\left[h\right]}=T]=P[T_{\infty ,\circ }^{\left[h\right]}=T]\cdot \frac{E\eta }{N-t}\cdot P\left[D+\sum_{j=t+1}^{N}\xi_{j}=N-1\right].$$

The local central limit theorem for the sum of independent and identically distributed random integers yields that

$$P\left[D+\sum_{j=t+1}^{N}\xi_{j}=N-1\right]=(1+o(1))\frac{k\gcd(\Omega_{\text{out}})}{\sqrt{2\pi NV\text{ar}[\xi ]}}$$

which implies

(40) $$\begin{array}{ll} P[|T_{\circ }|=N,T_{\circ }^{\left[h\right]}=T]=(1+o(1))\;P[T_{\infty ,\circ }^{\left[h\right]}=T]\;n^{-3/2}\;\frac{E\eta \gcd(\Omega_{\text{out}})}{\sqrt{2\pi kV\text{ar}\xi }}. & \; \end{array}$$

Let *d*(*o*) denote the root‐degree of *T*_∘_. It holds, since *ζ* has finite exponential moments, that $P[\eta \geq \log{\left(n\right)}^{2}]$ is exponentially small. Hence, using the cycle lemma and the central local limit theorem in an identical fashion as above, it follows that

$$\begin{array}{ll} P[|T_{\circ }|=N] & =o(n^{-3/2})+\sum_{d=1}^{\log{\left(n\right)}^{2}}P[\eta =d]\frac{d}{N-1}P[d+\sum_{j=2}^{N}\xi_{j}=N-1]\;\\ & =(1+o(1))n^{-3/2}E\eta \frac{\gcd(\Omega_{\text{out}})}{\sqrt{2\pi kV\text{ar}\xi }}, \end{array}$$

which, together with (40), implies (37), which completes the proof.

We are now finally in the position to complete the proof of our second main theorem.

Proof of Theorem 2 Let *ℓ* be an integer and let *G* be an arbitrary finite unlabelled Ω‐*k*‐tree that is rooted at a front. We claim that there exist an integer *L* ≥ 0, that depends on both *ℓ* and *G*, and a set E of finite plane trees, such that any plane tree *T*, that corresponds to a (*k*,Ω)‐coding tree *ψ*(*T*) and hence to a front‐rooted Ω‐*k*‐tree *G*(*T*): = *ϕ* ^−1^(*ψ*(*T*)), has the property *U*_*ℓ*_(*G*(*T*)) = *G* if and only if $T^{\left[L\right]}\in E$.

Before we prove the claim, we show that Theorem 2 is a direct consequence of this claim. By Lemma 8 we have

$$\lim_{n\to \infty }P[T_{n,\circ }^{\left[L\right]}\in E]=P[T_{\infty ,\circ }^{\left[L\right]}\in E]$$

and consequently

$$\lim_{n\to \infty }P[U_{\ell }(G_{n,k}^{\circ })=G]=P[U_{\ell }(G_{\infty ,k})=G],$$

where *G*_*∞*,*k*_ denotes the Ω‐*k*‐tree corresponding to *T*_*∞*,∘_, as desired.

In order to prove the existence of such an integer *L* and a set E we argue as follows. To each plane tree *T* we associate a unique sequence of increasing subtrees *T*_0_,*T*_1_,… of *T* that all contain the root‐vertex of *T* and have the property *G*(*T*_*i*_) = *U*_*i*_(*G*(*T*)) for all *i*. Of course, the tree *T*_*ℓ*_ may, in general, have arbitrarily large height. However, in order to satisfy *G*(*T*_*ℓ*_) = *G*, the tree *T*_*ℓ*_ may not have more vertices than the number of fronts in *G*. In particular, the height of *T*_*ℓ*_ is bounded by the number of fronts of *G*. Hence there exists a finite integer *L* such that for every plane tree *T* we may decide whether *U*_*ℓ*_(*G*(*T*)) = *G* is true or not just by looking at *T* ^[*L*]^.

## ACKNOWLEDGEMENTS

We would like to thank the anonymous reviewers for their very careful reviews of our paper, and for the comments, the corrections and suggestions that lead to a significantly improved paper. We also thank Charles Burnette (Academia Sinica) for his proofreading. The first author is partially supported by the Austrian Science Fund FWF, Project SFB F50‐02. The second author was supported by the Austrian Research Fund FWF, Project SFB F50‐03/02 and is supported by FWF‐MOST (Austria‐Taiwan) project I 2309‐N35. The third author is supported by the German Research Foundation DFG, STU 679/1‐1.
